# Probing the Lewis Acidity of Boronic Acids through Interactions with Arene Substituents

**DOI:** 10.1002/chem.202104044

**Published:** 2022-01-22

**Authors:** Jie Jian, Roel Hammink, Christine J. McKenzie, F. Matthias Bickelhaupt, Jordi Poater, Jasmin Mecinović

**Affiliations:** ^1^ Department of Physics, Chemistry and Pharmacy University of Southern Denmark Campusvej, 55 5230 Odense Denmark; ^2^ Division of Immunotherapy Oncode Institute Radboud University Medical Center 6525 GA Nijmegen The Netherlands; ^3^ Department of Tumor Immunology Radboud Institute for Molecular Life Sciences Radboud University Medical Center Geert Grooteplein 26 6525 GA Nijmegen The Netherlands; ^4^ Institute for Molecules and Materials Radboud University Heyendaalseweg 135 6525 AJ Nijmegen The Netherlands; ^5^ Department of Theoretical Chemistry Amsterdam Center for Multiscale Modeling Vrije Universiteit Amsterdam De Boelelaan 1083 1081 HV Amsterdam The Netherlands; ^6^ ICREA Passeig Lluís Companys 23 08010 Barcelona Spain; ^7^ Departament de Química Inorgànica i Orgànica & IQTCUB Universitat de Barcelona Martí i Franquès 1-11 08028 Barcelona Spain

**Keywords:** aromatic compounds, boronic acids, Lewis acidity, non-covalent interactions, polar-π interactions

## Abstract

Boronic acids are Lewis acids that exist in equilibrium with boronate forms in aqueous solution. Here we experimentally and computationally investigated the Lewis acidity of 2,6‐diarylphenylboronic acids; specially designed phenylboronic acids that possess two flanking aromatic rings with tunable aromatic character. Hammett analysis of 2,6‐diarylphenylboronic acids reveals that their Lewis acidity remains unchanged upon the introduction of EWG/EDG at the distant *para* position of the flanking aromatic rings. Structural and computational studies demonstrate that polar‐π interactions and solvation effects contribute to the stabilization of boronic acids and boronate forms by aromatic rings. Our physical‐organic chemistry work highlights that boronic acids and boronates can be stabilized by aromatic systems, leading to an important molecular knowledge for rational design and development of boronic acid‐based catalysts and inhibitors of biomedically important proteins.

## Graphical abstract

**Boronic acids**. Hammett analysis of 2,6‐diarylphenylboronic acids reveals that their Lewis acidity remains unchanged upon the introduction of EWG/EDG at the distant *para* position of the flanking aromatic rings. Structural and computational studies demonstrate that polar‐π interactions and solvation effects contribute to the stabilization of boronic acids and boronate forms by aromatic rings, providing a valuable molecular insight into the rational design of novel catalysts and inhibitors.

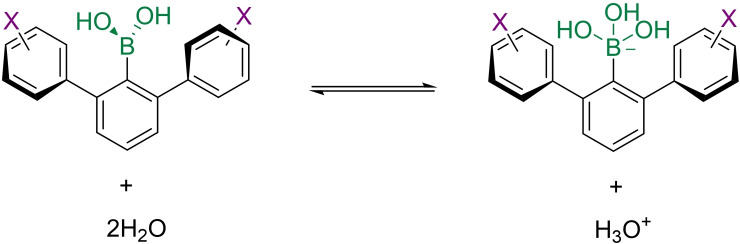

## Introduction

**Figure 1:**
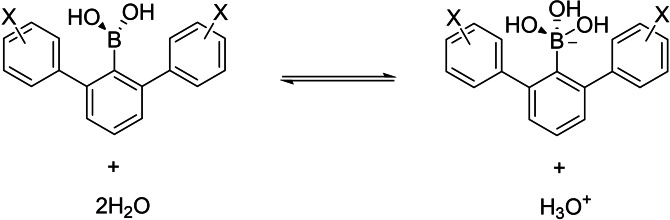
Dissociation of 2,6‐Diarylphenylboronic acids in water.

Boronic acids represent a class of organic molecules that have found a widespread use in molecular sciences, ranging from synthetic organic and supramolecular chemistry to medicinal chemistry and chemical biology.^[1]^ Perhaps best known as common starting materials in Suzuki cross‐coupling reactions, boronic acids react with organohalides to enable the construction of simple and more complex molecular frameworks.^[2]^ A growing body of recent work has also demonstrated that boronic acids can be utilized in catalysis and dynamic combinatorial chemistry as a result of reversible covalent bonds involving the hydroxy groups.^[3]^ Beyond organic chemistry, boronic acids display unique chemical properties as inhibitors that specifically bind to proteins, most notably as reversible covalent inhibitors targeting nucleophilic residues in proteins’ active sites.^[4]^ Despite their numerous roles in chemistry, it is presently poorly understood whether, and how, boronic acids interact with common functional groups. Due to the presence of the empty p_z_ orbital of boron, boronic acids act as Lewis acids, interacting with water, anions and electron‐rich functionalities.^[3d]^ The two nucleophilic hydroxy groups can also participate in hydrogen bonding, both as hydrogen bond donors and acceptors.^[5]^ In this work, we investigate whether boronic acids and their boronate forms have an ability to interact with aromatic rings. Aromatic rings have been extensively studied in their ability to stabilize polar functionalities, including cations, anions and neutral groups, both in chemical and biomolecular systems.^[6]^ We use specially designed 2,6‐diarylphenylboronic acids as a model system for investigations of intramolecular polar‐π interactions between the central boronic acid/boronate moiety and the two neighboring flanking aromatic rings (Figure 1). Related scaffolds have been employed for probing other types of through‐space polar‐π interactions in carboxylic acids, anilines, phenols, thiophenols, sulfonamides and tetrazoles.^[7]^

## Results and Discussion

**Scheme 1:**
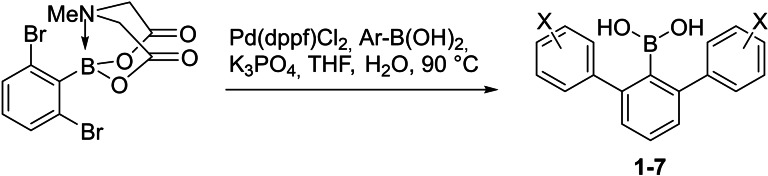
Synthesis of boronic acids **1**–**7**.

To experimentally and computationally examine the involvement of boronic acid and boronate forms in association with electron‐rich aromatic rings in 2,6‐diarylphenylboronic acids, we introduced electron‐donating and electron‐withdrawing substituents on the *para*/*meta*‐position of flanking aromatic rings, which enabled the strength of non‐covalent interactions and Lewis acidity to be probed. Substituted 2,6‐diarylphenylboronic acids **1**–**7** were synthesized from the *N*‐methyliminodiacetic acid (MIDA) ester of 2,6‐dibromophenylboronic acid via palladium‐catalyzed Suzuki coupling with *para*/*meta*‐substituted phenylboronic acid (Scheme 1). The MIDA ester was hydrolyzed simultaneously under mildly basic conditions to offer the free 2,6‐diarylphenylboronic acid.

**Figure 2:**
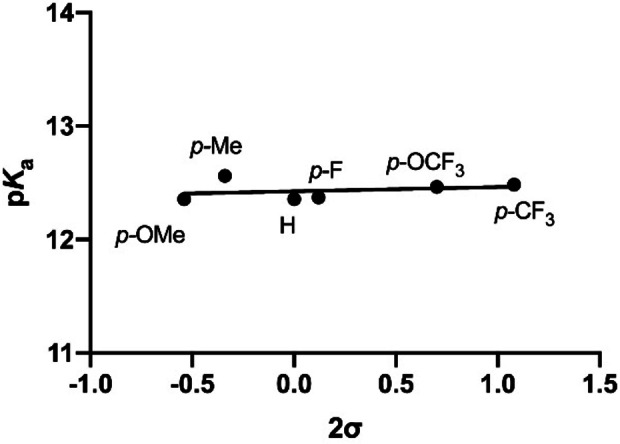
Correlation between p*K*_a_ values of boronic acids **1**–**6** and the Hammett sigma values (2σ).

Measurements of p*K*_a_ values of boronic acids **1**–**7** in buffered water and acetonitrile (in 3 : 1 ratio to guarantee that all compounds are fully soluble) by UV‐Vis spectroscopy showed that all boronic acids have very similar strength of Lewis acidity (p*K*_a_∼12.4, Table 1 and Figure S1 in Supporting Information). For *para*‐substituted boronic acids **1**–**6**, correlations between p*K*_a_ values and Hammett sigma values (2σ, due to the presence of two flanking aromatic rings) revealed a flat plot with virtually no dependence of p*K*_a_ values on 2σ values (slope=0.04, ρ=−0.04) (Figure 2 and Figure S1). These results are markedly different to those obtained from related 2,6‐diaryl aromatic systems on Brønsted acids/bases,^[7]^ in which there was a pronounced linear trend with large positive ρ values. Overall, our observations indicate that boronic acids are not stabilized by electron‐rich aromatic rings (e. g., with *para*‐OMe) more than they are stabilized by electron‐poor aromatic rings (e. g., with *para*‐CF_3_), as both extreme cases have very similar p*K*_a_ values (12.36 for *para*‐OMe and 12.49 for *para*‐CF_3_). Comparison of fluoro‐substituted boronic acids **4** and **7** that have different σ values, but similar p*K*_a_ values indicates that the through‐space, and not the through‐bond, effect plays a major role in their stabilization. The experimentally measured similar p*K*_a_ values across the series of 2,6‐diarylphenylboronic acids **1**–**7** suggest that the underlying mechanism for stabilization of boronic acids/boronates relies on well‐balanced contributions from two opposing effects that stabilize the boronic acid form (i. e., the Lewis acid) and the boronate form (i. e., the conjugate base). In addition, to further investigate the effect of solvent on the Lewis acidity of boronic acids, we measured p*K*_a_ values of phenylboronic acid and 4‐methoxyphenylboronic acid, two water soluble boronic acids. Both boronic acids displayed a more pronounced acidity in water than in water/acetonitrile mixture (p*K*_a_ for phenylboronic acid is 8.68 vs. 9.61, p*K*_a_ for 4‐methoxyphenylboronic acid is 9.25 vs. 10.29, Figure S2, Table S1).^[8]^ Overall, these values indicate that 2,6‐diarylphenylboronic acids are weaker acids than unsubstituted phenylboronic acid, a result that we attribute to steric effects commonly observed in related *ortho*‐substituted aromatic systems.[^7b^, ^7d^, ^9^]

**Table 1:** p*K*_a_ values for boronic acids **1**–**7**.

compd	X	*σ*	p*K*_a_^[a]^
1	H	0.00	12.36
2	*p*‐OMe	−0.27	12.36
3	*p*‐Me	−0.17	12.56
4	*p*‐F	0.06	12.37
5	*p‐*OCF_3_	0.35	12.47
6	*p*‐CF_3_	0.54	12.49
7	*m*‐F	0.34	12.38

[a] Determined in H_2_O/acetonitrile=3 : 1.

**Figure 3:**
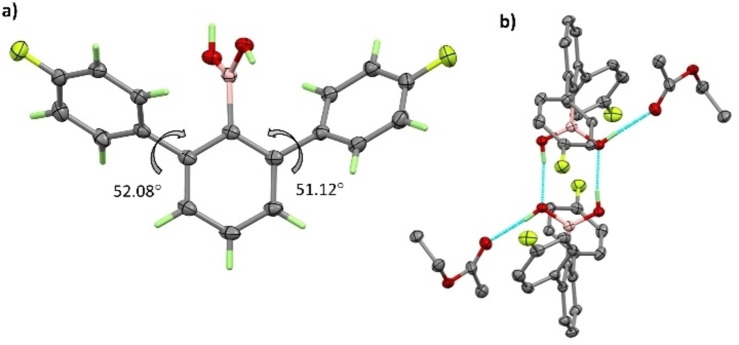
(a) The X‐ray structure of 2,6‐diarylphenylboronic acid **4** showing the dihedral angles between the planes of the flanking rings relative to the central ring. Non‐hydrogen atoms are drawn with 50 % probability ellipsoids. (b) Intermolecular H‐bonding arrangements with only the participating H atoms shown.

The single‐crystal X‐ray structure of the 2,6‐diarylphenylboronic acid **4** co‐crystallized with ethyl acetate is consistent with the solution state results in showing no evidence of intramolecular BOH‐π interactions (Figure 3a and Figure S3). Instead, both OH groups are involved in strong intermolecular H‐bonding. Pairs of adjacent molecules form H‐bonded dimers where one H atom (H2) from each boronic acid group participates (Figure 3b). The BO…HOB distance is 1.89 Å. The boronic acid H atom not involved in the intradimer bonding is H‐bonded to the carbonyl group of the co‐crystallized ethyl acetate molecule with BOH⋅⋅⋅O=C, 1.89 Å. The flanking aromatic rings are not coplanar. Relative to the central phenylboronic acid ring the dihedral angles with the flanking rings are 52.08° and 51.12° as shown in Figure 3a. These rings are staggered when viewing parallel to the plane of the central ring. The crystal structure of the methyl ester derivative of **3**, **3^Me^**, has also been determined (Figure S4) and shows that relative orientations of the aryl rings are similar to those in **4**.

**Figure 4:**
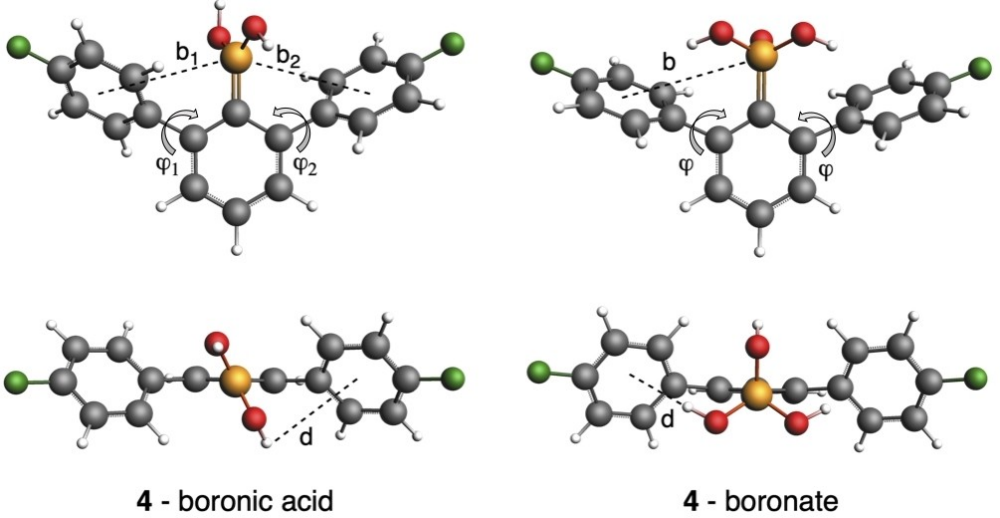
Equilibrium staggered geometry of boronic acid **4** and its boronate form in eclipsed conformation. Distance OH⋅⋅⋅center of aryl ring (d), distance B⋅⋅⋅center of the aryl ring (b), and dihedral angles (ϕ) are enclosed. Computed at ZORA‐BLYP‐D3(BJ)/TZ2P in water.

Building on quantum chemical analyses of related 2,6‐diaryl aromatic systems,[^7b^, ^7d^, ^7e^, ^7f^, ^7g^] we applied the same computational analysis to understand the interactions between boronic acid/boronate forms and aromatic ring in the series of 2,6‐diarylphenylboronic acids at the ZORA‐BLYP‐D3(BJ)/TZ2P level in aqueous solvation with ADF^[10]^ with the aim to reveal the underlying electronic mechanism for the through‐space polar‐π interactions by means of an energy decomposition analysis (EDA) in both boronic acid and boronate forms. The staggered conformation of 2,6‐diarylphenylboronic acids is more stable than the eclipsed conformation by up to 2.2 kcal mol^−1^ in aqueous solution (Figure 4 and Tables S2‐S4). The distance between the H of the hydroxyl and the center of the aryl rings is between 3.55 and 3.61 Å, with both OH groups pointing away from aromatic rings, in line with crystallographic data. Such a small difference is further supported by the rotational barrier (Figure S5) calculated for 2,6‐diarylphenylboronic acid **1**, where the conformers in the range ϕ_1_=30–120° differ by less than 2 kcal mol^−1^. In contrast, the equilibrium structure for the boronate form is the eclipsed conformation due to the steric repulsion caused by the introduction of a third hydroxyl group. Now, the distance between the H of the hydroxyl and the center of the aromatic ring is not only much shorter (2.53–2.56 Å), but these H point towards the center (Figure 4).

Table 2 lists the boronate formation energies (Δ*E* ^*boronate*^) of the 2,6‐diarylphenylboronic acids. The Δ*E* values are calculated from the reaction ArB(OH)_2_+2H_2_O→ArB(OH)_3_^−^+H_3_O^+^. For completeness, the analyzed Lewis acidity was also compared to the possible Brønsted acidity of 2,6‐diarylphenylboronic acids. Indeed, we find that 2,6‐diarylphenylboronic acids are not Brønsted acids, as reflected by the more than 150 kcal mol^−1^ higher computed proton affinities for these systems, that is, energy changes associated with the simple heterolytic dissociation of a proton: ArB(OH)_2_→ArB(OH)O^−^+H^+^ (Table S6). We find that the computed Δ*E* ^*boronate*^ values in water vary only slightly as a function of 2σ (Figure S6), in agreement with the experimental p*K*_a_ values, which remain constant across the series. The fact that both *p*‐F (**4**) and *m*‐F (**7**) systems present similar Δ*E* ^*boronate*^ values provides further support of the presence of through‐space interactions in this series of 2,6‐diarylphenylboronic acids.

**Table 2:** Boronate formation energies of the 2,6‐diarylphenylboronic acids in water, together with solvation energies of boronic acid+2H_2_O, and boronate+H_3_O^+^, and the corresponding differential solvation (in kcal mol^−1^).^[a]^

compd	X	Δ*E* ^*boronate*^	Δ*E*_solv_^boronic+2H2O^	Δ*E*_solv_^boronate+H3O+^	ΔΔ*E*_solv_
1	H	47.1	−19.1	−142.8	−123.6
2	*p*‐OMe	47.8	−22.4	−148.7	−126.3
3	*p*‐Me	47.4	−18.9	−143.6	−124.7
4	*p*‐F	47.1	−21.1	−139.7	−118.7
5	*p*‐OCF_3_	47.3	−22.3	−135.3	−113.0
6	*p*‐CF_3_	47.0	−22.5	−134.1	−111.7
7	*m*‐F	47.0	−20.9	−139.9	−118.9

[a] Δ*E* ^*boronate*^ of the 2,6‐diarylphenylboronic acids in vacuo enclosed in Table S5.

**Figure 5:**
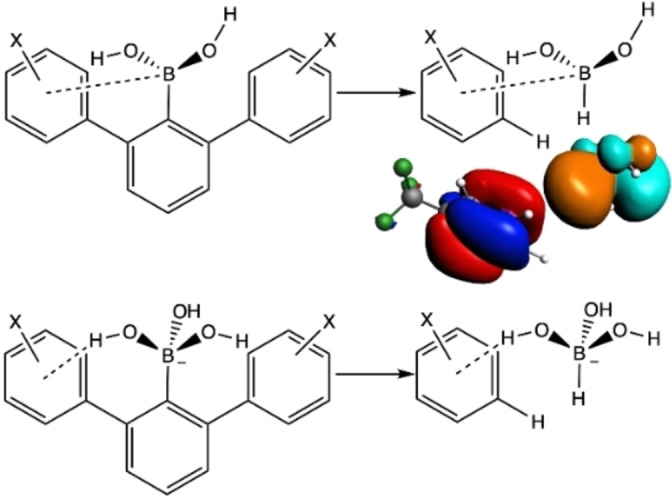
Model systems used for the analysis of through‐space BOH‐π interactions in 2,6‐diarylphenylboronic acid (top) and its boronate (bottom) in water. Interacting HOMO π orbital of aryl and LUMO empty p orbital of B of boronic acid are also included.

Through‐space polar‐π interactions in the boronic acid and the boronate systems were analyzed by means of an EDA to unravel their nature and, especially, to understand how they result in essentially constant Δ*E* ^*boronate*^ as function of arylic substitution. These analyses reveal that variation of the arylic substituent does modify the intrinsic interaction in the boronate systems and, thus, the gas phase Δ*E* ^*boronate*^. However, as we will explain, the resulting trend in gas phase Δ*E* ^*boronate*^ is nearly canceled by a counteracting trend in solvent effects. Thus, we analyzed a truncated model system, in which the phenyl group linked to B(OH)_2_ has been substituted by a H atom, and only one aryl group has been kept (Figure 5). The geometries and relative position and orientation of both the HB(OH)_2_ group and the aryl ring are exactly those of the equilibrium structure. The interaction between HB(OH)_2_ and the aromatic ring is slightly destabilizing caused by the Pauli repulsion term due to the close proximity between the two fragments (B−H of the boronic acid and C−H of the aryl) (Tables S7 and S8). This slight destabilization in Δ*E*_int_ when going from EDG to EWG is due to the weakening in the B⋅⋅⋅π interaction as the π‐system on the ring becomes less electron rich, thus causing a less attractive Δ*V*_elstat_ term as well as a slightly less attractive Δ*E*_oi_ term. The relative weakness of this through‐space interaction originates from the long distance between the boron group and the aryl ring, causing the overlap and interaction between the empty boron 2p_z_ acceptor orbital and the π‐electron system on the aromatic ring to be weak.

If the same approach is applied to the boronate form, the net interaction Δ*E*_int_ between the two fragments, that is, HB(OH)_3_^−^ and the substituted aryl ring, becomes stabilizing. The strength of this stabilizing BOH⋅⋅⋅π interaction increases from *p*‐OMe to *p*‐CF_3_ (from 0.1 to −5.6 kcal mol^−1^, Tables S7 and S8). This is so, despite the fact that the Pauli repulsion is larger for the boronates than for the boronic acids, and because of the closer proximity of the hydroxyl to the aryl ring. The reason that the net interaction Δ*E*_int_ turns from slightly destabilizing to stabilizing is the more favorable electrostatic and orbital interactions. In particular, Δ*V*_elstat_ becomes more attractive in the case of EWG due to the fact that the electron density of the net negatively charged boronate form remains involved in an essentially constant and significantly stabilizing interaction with the nuclei of the aryl group, whereas its repulsion with the electron density on the aryl group diminishes by going from EDG to EWG.

The above bonding analyses show that the intrinsic (vacuum) through‐space polar‐π interactions increasingly stabilize the boronate bases, that is, the gas‐phase Δ*E* ^*boronate*^ clearly decreases, as the arylic substituent becomes more electron‐withdrawing. Why then is the boronate formation energy of boronic acids hardly affected by varying these substituents, both in our experiments and in the earlier discussed computations? The answer is: differential solvation of the anionic boronate+the oxonium ion *versus* the neutral boronic acid+water: ΔΔ*E*_solv_=Δ*E*_solv_(boronate+H_3_O^+^)−Δ*E*_solv_(boronic acid+2H_2_O) (Table 2). Thus, the solvation energy of the boronic acid form, that is, Δ*E*_solv_(boronic acid+2H_2_O), is 18.9 to 22.5 kcal mol^−1^ less stabilizing than that for the charged boronate, that is, Δ*E*_solv_(boronate+H_3_O^+^). The latter pronouncedly weakens, from −148.7 to −134.1 kcal mol^−1^ as we go from *p*‐OMe to *p*‐CF_3_ and, thus, the differential solvation (ΔΔ*E*_solv_ in Table 2) also decreases, namely, from −126.3 to −111.7 from *p*‐OMe to *p*‐CF_3_. This means that the boronate form is much less stabilized by solvation in the case of more electron‐withdrawing groups.

What is the reason of this weaker solvation of the boronate form when going from EDG to EWG? Back to our model system, it is shown how the larger ΔΔ*E*_solv_ for *p*‐CF_3_ correlates with the larger charge transfer from the boronate fragment to the aryl ring when we have an EWG (Table 3). For example, the charge on the HB(OH)_3_^−^ fragment is −0.893 and −0.868 a.u. for *p*‐OMe and *p*‐CF_3_ substituents, respectively. Such charge transfer causes the net charge to be more spread across the entire system and, thus, it is less stabilized by solvation. This is reflected by the solvation energy of the complex, Δ*E*_solv_^comp^, which is significantly more stabilizing in the boronate than in the boronic acid and, importantly, which weakens from *p*‐OMe to *p*‐CF_3_. This loss of solvation stabilization of the boronate, as we go from *p*‐OMe to *p*‐CF_3_ substituents, counteracts and essentially cancels the simultaneous gain in intrinsic (vacuum) through‐space polar‐π interaction. Consequently, the through‐space polar‐π interactions in aqueous solution, (e. g., 3.28 vs. 3.26 kcal mol^−1^ for *p*‐OMe and *p*‐CF_3_) and, thus, the Δ*E* ^*boronate*^ and p*K*_a_ values in aqueous solution (e. g., 12.36 vs. 12.49 for *p*‐OMe and *p*‐CF_3_, Table 1) remain approximately constant as function of the Hammett sigma values.

**Table 3:** Interaction energy in vacuo (Δ*E*_int_^gas^) and in aqueous solvation (Δ*E*_int_^aq^), and solvation of complex energy (Δ*E*_solv_^comp^) (in kcal mol^−1^) corresponding to the interaction between the B(OH)_2_ group and the π‐ring in the model system (Figure 5) derived from the 2,6‐diarylphenylboronic acids and their boronate form. Charge on functional group fragment (in a.u.) is also enclosed.^[a,b]^

	X	Δ*E*_int_^aq^	Δ*E*_int_^gas^	Δ*E*_solv_^comp^	q_B group_
boronic acid	*p*‐OMe	2.71	2.04	−9.30	−0.013
	*p*‐CF_3_	2.78	2.71	−9.50	−0.002
boronate	*p*‐OMe	3.28	0.14	−73.07	−0.893
	*p*‐CF_3_	3.26	−5.57	−66.60	−0.868

[a] Δ*E*_int_^aq^=Δ*E*_int_^gas^−Δ*E*_desolv_^frag^+Δ*E*_solv_^comp^. [b] See complete Table and the rest of systems enclosed in Table S9.

## Conclusion

Understanding the reactivity of boronic acids is central for development of novel catalytic reactions, dynamic molecular systems and inhibitors. Despite well‐established role of boronic acids in molecular sciences, detailed examinations of their Lewis acidity via through‐space effects have not been carried out. In the present study, we have used a specially designed 2,6‐diarylphenylboronic acid scaffold for detailed molecular examinations of non‐covalent interactions between aromatic rings and neighboring boronic acids and boronates as Lewis acids and conjugate bases, respectively. This molecular architecture allowed us to precisely probe the dependence of Lewis acidity of boronic acids on Hammett sigma values of substituents on the flanking aromatic rings, supported by structural and advanced computational analyses. Our physical‐organic study presented here demonstrates that 2,6‐diarylphenylboronic acids and their boronate forms are stabilized by the flanking aromatic rings via through‐space polar‐π interactions, whereas solvation effects also contribute to constant Lewis acidity strength across the series of 2,6‐diarylphenylboronic acids that possess electron‐donating or electron‐withdrawing substituents at the *para*‐position of the flanking rings. Computations reveal that due to very high calculated proton affinities 2,6‐diarylphenylboronic acids do not display pronounced Brønsted acidity. Our structural and computational work importantly shows that, unlike Brønsted acids,^[7]^ boronic acids do not interact with aromatic rings via energetically favorable OH‐π interactions. These results are important both for designing new boronic acid‐based catalysts and rational drug design that rely on non‐covalent interactions between aromatic rings and polar boronic acid and boronate functionalities. Our work further justifies and expands the use of 2,6‐diaryl aromatic systems for detailed examinations of through‐space interactions involving aromatic rings.^[7]^

## Experimental Section

**General Experimental Procedures**: Melting points were measured on an Buchi 535 melting point apparatus. ^1^H, ^13^C and ^19^F NMR spectra were obtained using Bruker Avance III 400 MHz NMR spectrometer. ^11^B NMR spectra were measured using JEOL JNM‐ECZR 500 MHz spectrometer. High‐resolution mass spectra (HRMS) were obtained on a Bruker Daltonics‐micrOTOF‐Q II‐ESI‐Qq‐TOF mass spectrometer. The microwave reactions were performed on Biotage Initiator+ microwave synthesizer. Chemical shifts are reported in parts per million (ppm δ) referenced to the residual ^1^H resonance of the solvent (CDCl_3_, 7.26 ppm) and the residual ^13^C resonance of the solvent (CDCl_3_, 77.16 ppm). Splitting patterns are designated as follows: s, singlet; d, doublet; dd, doublet of doublets; ddd, doublet of doublet of doublets; tdd, triplet of doublet of doublets; t, triplet; q, quartet; m, mulitplet. Coupling constants (*J*) are reported in Hertz (Hz). All reagents and solvents were purchased from commercial sources and used without further purification.

**Synthesis of 2,6‐diarylphenylboronic acids**: To a mixture of 2,6‐dibromophenylboronic acid MIDA ester (60 mg, 0.154 mmol) in THF, arylboronic acids (0.383 mmol, 2.5 equiv.), K_3_PO_4_ (130 mg, 0.615 mmol, 4 equiv.) and H_2_O (19 μL, 1.078 mmol, 7 equiv.) were added. After it was heated at 90 °C under microwave for 6 h, the reaction mixture was poured into 5 mL of water and extracted with ethyl acetate (3×10 mL). The combined organic layers were washed with brine, dried over MgSO_4_, filtered and concentrated. The residue was purified by flash column chromatography on silica gel (PE/EA) to give the 2,6‐diarybenzeneboronic acid.

**2,6‐Diphenylphenylboronic acid** (***1***): White solid (8 mg, 19 %); mp 143–145 °C; ^1^H NMR (400 MHz, CDCl_3_) δ 7.54–7.34 (m, 13H), 4.13 (s, 2H); ^13^C{^1^H} NMR (101 MHz, CDCl_3_) δ 146.1, 143.3, 129.2, 128.7, 128.1, 127.6; ^11^B NMR (160 MHz, CDCl_3_) δ 30.48; HRMS (ESI): *m/z* calcd for C_18_H_15_BNaO_2_ [M+Na]^+^: 297.1061, found 297.1065.

**2,6‐Di(4‐methoxy)phenylphenylboronic acid** (***2***): Yellowish oil (12 mg, 23 %); ^1^H NMR (400 MHz, CDCl_3_) δ 7.45 (dd, *J*=8.3, 7.0 Hz, 1H), 7.41–7.36 (m, 4H), 7.35–7.31 (m, 2H), 6.99–6.92 (m, 4H), 4.23 (s, 2H), 3.84 (s, 6H); ^13^C{^1^H} NMR (101 MHz, CDCl_3_) δ 159.3, 145.6, 135.7, 129.8, 129.1, 127.7, 114.1, 55.4; ^11^B NMR (160 MHz, CDCl_3_) δ 30.19; HRMS (ESI): *m/z* calcd for C_20_H_19_BNaO_4_ [M+Na]^+^: 357.1272, found 357.1281.

**2,6‐Di(4‐methyl)phenylphenylboronic acid** (***3***): White solid (20 mg, 43 %); mp 156–157 °C; ^1^H NMR (400 MHz, CDCl_3_) δ 7.47 (dd, *J*=8.3, 6.9 Hz, 1H), 7.39–7.33 (m, 6H), 7.26–7.22 (m, 4H), 4.16 (s, 2H), 2.41 (s, 6H); ^13^C{^1^H} NMR (101 MHz, CDCl_3_) δ 146.0, 140.4, 137.3, 129.4, 129.1, 128.6, 127.9, 21.3; ^11^B NMR (160 MHz, CDCl_3_) δ 30.62; HRMS (ESI): *m/z* calcd for C_20_H_19_BNaO_2_ [M+Na]^+^: 325.1374, found 325.1386.

**2,6‐Di(4‐fluoro)phenylphenylboronic acid** (***4***): White solid (16 mg, 34 %); mp 172 °C; ^1^H NMR (400 MHz, CDCl_3_) δ 7.47 (dd, *J*=8.2, 7.1 Hz, 1H), 7.44–7.39 (m, 4H), 7.35 (d, *J*=7.6 Hz, 2H), 7.15–7.07 (m, 4H), 4.23 (s, 2H); ^13^C{^1^H} NMR (101 MHz, CDCl_3_) δ 162.6 (d, *J*=246.7 Hz), 144.9, 139.2 (d, *J*=3.2 Hz), 130.3 (d, *J*=8.1 Hz), 129.3, 128.1, 115.6 (d, *J*=21.5 Hz); ^19^F NMR (376 MHz, CDCl_3_) δ −115.1; ^11^B NMR (160 MHz, CDCl_3_) δ 30.51; HRMS (ESI): *m/z* calcd for C_18_H_13_BF_2_NaO_2_ [M+Na]^+^: 333.0872, found 333.0882.

**2,6‐Di(4‐trifluoromethoxyl)phenylphenylboronic acid** (***5***): White solid (12 mg, 18 %); mp 162 °C; ^1^H NMR (400 MHz, CDCl_3_) δ 7.53–7.44 (m, 5H), 7.37 (d, *J*=7.6 Hz, 2H), 7.31–7.23 (m, 4H), 4.22 (s, 2H); ^13^C{^1^H} NMR (101 MHz, CDCl_3_) δ 149.0, 144.6, 141.7, 130.1, 129.4, 128.4, 121.9, 121.0, 119.4; ^19^F NMR (376 MHz, CDCl_3_) δ −57.7; ^11^B NMR (160 MHz, CDCl_3_) δ 30.24; HRMS (ESI): *m/z* calcd for C_20_H_13_BF_6_NaO_4_ [M+Na]^+^: 465.0707, found 465.0704.

**2,6‐Di(4‐trifluoromethyl)phenylphenylboronic acid** (***6***): Yellowish solid (17 mg, 27 %); mp 144–147 °C; ^1^H NMR (400 MHz, CDCl_3_) δ 7.73–7.66 (m, 4H), 7.63–7.57 (m, 4H), 7.54 (dd, *J*=8.3, 7.1 Hz, 1H), 7.44–7.40 (m, 2H), 4.26 (s, 2H); ^13^C{^1^H} NMR (101 MHz, CDCl_3_) δ 146.6, 144.7, 130.1, 129.8, 129.5, 129.1, 128.7, 125.70 (q, *J*=3.8 Hz), 123.0; ^19^F NMR (376 MHz, CDCl_3_) δ −62.4; ^11^B NMR (160 MHz, CDCl_3_) δ 30.24; HRMS (ESI): *m/z* calcd for C_20_H_13_BF_6_NaO_2_ [M+Na]^+^: 433.0809, found 433.0805.

**2,6‐Di(3‐fluoro)phenylphenylboronic acid** (***7***): White solid (20 mg, 42 %); mp 150 °C; ^1^H NMR (400 MHz, CDCl_3_) δ 7.50 (dd, *J*=8.3, 7.0 Hz, 1H), 7.42–7.36 (m, 4H), 7.24 (ddd, *J*=7.6, 1.7, 1.0 Hz, 2H), 7.18 (ddd, *J*=9.7, 2.6, 1.7 Hz, 2H), 7.08 (tdd, *J*=8.3, 2.6, 1.0 Hz, 2H), 4.24 (s, 2H); ^13^C{^1^H} NMR (101 MHz, CDCl_3_) δ 162.9 (d, *J*=246.8 Hz), 145.4 (d, *J*=7.5 Hz), 144.8, 144.8, 130.2 (d, *J*=8.4 Hz), 129.3, 128.4, 124.5 (d, *J*=2.9 Hz), 115.8 (d, *J*=21.7 Hz), 114.6 (d, *J*=21.0 Hz); ^11^B NMR (160 MHz, CDCl_3_) δ 30.24; ^19^F NMR (376 MHz, CDCl_3_) δ −112.7; HRMS (ESI): *m/z* calcd for C_18_H_13_BF_2_NaO_2_ [M+Na]^+^: 333.0872, found 333.0871.

**p*K***_**a**_ **measurements**: p*Ka* measurements of phenylboronic acids **1**–**7** were carried out with UV spectroscopy using a protocol described before.[^7b^, ^7e^] Briefly, a set of buffers covering a pH range of 3.6–13.5 was prepared. The pH range of 3.6–8.7 was covered by a citric acid‐sodium phosphate buffer. The range of 8.7–11 was covered by a borax/boronic acid buffer. The pH range of 11–13 was covered by a disodium hydrogen phosphate/sodium phosphate buffer. The pH range of 13–13.5 was covered by a KCl/KOH buffer. 25 ml of all buffers were adjusted to the right pH with 5 M NaOH or 9 M HCl and right ionic strength (0.1 M) using KCl, and the volume was adjusted to 50 ml. For the final pH values the solutions were measured again with 25 % acetonitrile added, since measurements are performed under these conditions. Next, boronic acids **1**–**7** were dissolved in acetonitrile at a concentration of 2.5 mM. Control compounds were dissolved in acetonitrile at a concentration of 2.5 mM and in water at a concentration of 2.5 mM. A 96‐well Microtiter Plate (UV star, Greiner Bio) was filled with buffers with increasing pH and in each well 40 ml acetonitrile (40 ml water in case of control compounds), 150 ml buffer and 10 ml compound solution were pipetted. Measurements were performed using a Tecan Spark M10 plate reader recorded in the range between 200–400 nm with a 2 nm resolution. Following the protocol from literature, the outcoming data was normalized and corrected and the spectral differences between the maximal and minimal spectra were plotted against the log (pH) (Figure S1, Figure S2 for control compounds). Finally, the pKa was determined using a 4‐parameter fit in Prism GraphPad 6.

**Single‐crystal X‐ray crystallography**: The data were collected at 100(1)K on a Synergy, Dualflex, AtlasS2 diffractometer using Cu_Kα_ radiation (λ=1.54184 Å) and the CrysAlis PRO 1.171.40.29a suite.^[11]^ Using SHELXLE^[12]^ and Olex2^[13]^ the structure was solved by dual space methods (SHELXT)^[14]^ and refined on *F* ^2^ using all the reflections (SHELXL‐2018/3).^[15]^ All the non‐hydrogen atoms were refined using anisotropic atomic displacement parameters and hydrogen atoms bonded to carbon inserted at calculated positions using a riding model. The ethoxy group (O4, C21 and C22) of the co‐crystallised ethyl acetate was modelled with 50 % occupancy over two positions (A and B). Brief summary for **4**: C_22_H_21_BF_2_O_4_ (*M* =398.20 g/mol): monoclinic, space group *P*2_1_/*c*, *a*=10.83640(10) Å, *b*=12.94010(10) Å, *c*=14.25270(10) Å, *β*=95.2170(10), *V*=1990.29(3) Å^3^, *Z*=4, *T*=100.00(14) K, *Dcalc*=1.329 g/cm^3^, 56100 reflections measured (8.194°≤2Θ≤149.274°), 4053 unique (*R*_int_=0.0234, *R*_sigma_=0.0086), which were used in all calculations. The final *R*_1_ was 0.0322 (I>2σ(I)) and *wR*_2_ was 0.0857 (all data). Brief summary for **3^Me^**: C_22_H_23_BO_2_ (*M* =330.21 g/mol): monoclinic, space group *P*2_1_/*c*, *a*=17.48630(10) Å, *b*=8.08000(10) Å, *c*=12.85930(10) Å, *β*=99.6320(10), *V*=1791.27(3) Å^3^, *Z*=4, *T*=100.00(14) K, *Dcalc*=1.224 g/cm^3^, 50412 reflections measured (5.126°≤2Θ≤149.19°), 3618 unique (*R*_int_=0.0479, *R*_sigma_=0.0159), which were used in all calculations. The final *R*_1_ was 0.0411 (I>2σ(I)) and *wR*_2_ was 0.1145 (all data). Crystals of **3^Me^** were obtained by crystallising compound **3** in methanol.

Deposition Number(s) 2078379 (for **4**) and 2078378 (for **3** ^**Me**^) contain(s) the supplementary crystallographic data for this paper. These data are provided free of charge by the joint Cambridge Crystallographic Data Centre and Fachinformationszentrum Karlsruhe Access Structures service.

**Quantum Chemical Analyses**: All calculations were carried out with the Amsterdam Density Functional (ADF) program using dispersion‐corrected density functional theory at the ZORA‐BLYP‐D3(BJ)/TZ2P level of theory.[^10^, ^16^] The effect of solvation in water was simulated by means of the Conductor like Screening Model (COSMO) of solvation as implemented in ADF. This level of theory has been previously shown to perform well for the computation of proton affinities,[^7b^, ^7d^, ^7e^, ^7f^, ^7g^] complexation energies of hydrogen bonds, and interaction energies of van der Waals and other weakly‐bonded complexes.[^3a^, ^17^]

The role of distance and geometry on through‐space interaction was analyzed within the framework of quantitative Kohn‐Sham molecular orbital theory in combination with a quantitative energy decomposition analysis (EDA) in the gas phase. The interaction energy Δ*E*_int_ between the boronic acid and aryl fragments was decomposed into the classical electrostatic attraction Δ*V*_elstat_, Pauli repulsion Δ*E*_Pauli_ between occupied orbitals, stabilizing orbital interactions Δ*E*_oi_, and dispersion Δ*E*_disp_ [Eq. 1]:^[18]^

(1) $$\Delta E{}_{\mathrm{int}}=\Delta V{}_{\mathrm{elstat}}+\Delta E{}_{\mathrm{Pauli}}+\Delta E{}_{\mathrm{oi}}+\Delta E{}_{\mathrm{disp}}$$

Herein, the term ΔV_elstat_ corresponds to the classical electrostatic interaction between the unperturbed charge distributions of the prepared (i. e., deformed). This term is usually attractive. The Pauli repulsion Δ*E*_Pauli_ results from the Pauli exclusion principle for fermions and comprises the destabilizing interactions between occupied orbitals and is responsible for the steric repulsion. And the orbital interaction Δ*E*_oi_ in any MO model, and therefore also in Kohn‐Sham theory, accounts for charge transfer (i. e., donor‐acceptor interactions between occupied orbitals on one moiety with unoccupied orbitals of the other, including the HOMO‐LUMO interactions) and polarization (empty/occupied orbital mixing on one fragment due to the presence of another fragment).

## Conflict of interest

The authors declare no conflict of interest.

1

## Supporting information

As a service to our authors and readers, this journal provides supporting information supplied by the authors. Such materials are peer reviewed and may be re‐organized for online delivery, but are not copy‐edited or typeset. Technical support issues arising from supporting information (other than missing files) should be addressed to the authors.

Supporting Information

## Data Availability

The data that support the findings of this study are available in the supplementary material of this article.
