# Supplementary material for: Probing the Lewis Acidity of Boronic Acids through Interactions with Arene Substituents
Source: Chemistry. 2022 Jan 22;28(9):e202104044. doi: 10.1002/chem.202104044 (PMC9306523; doi:10.1002/chem.202104044)
Supplement: Supplementary file 1 — Supporting Information [file CHEM-28-0-s001.pdf]

Chemistry–A European Journal

Supporting Information

Probing the Lewis Acidity of Boronic Acids through Interactions with Arene Substituents

Jie Jian, Roel Hammink, Christine J. McKenzie, F. Matthias Bickelhaupt, Jordi Poater,* and Jasmin Mecinović*

Table of Contents

1. NMR spectra..2

2. pK_a measurements..16

3. Single crystal X-ray crystallography..18

4. Quantum Chemical Analyses..19

1. NMR spectra

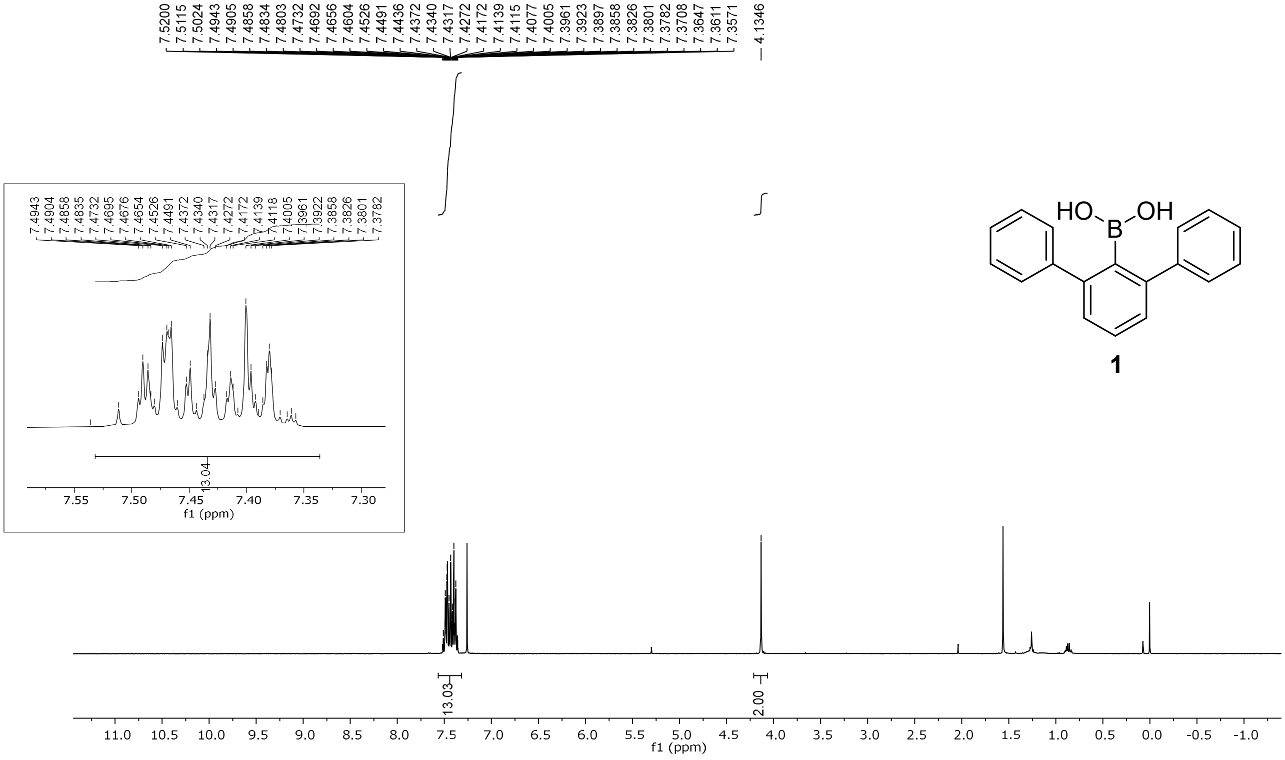

^{1}H NMR (400 MHz, CDCl3) of compound **1**

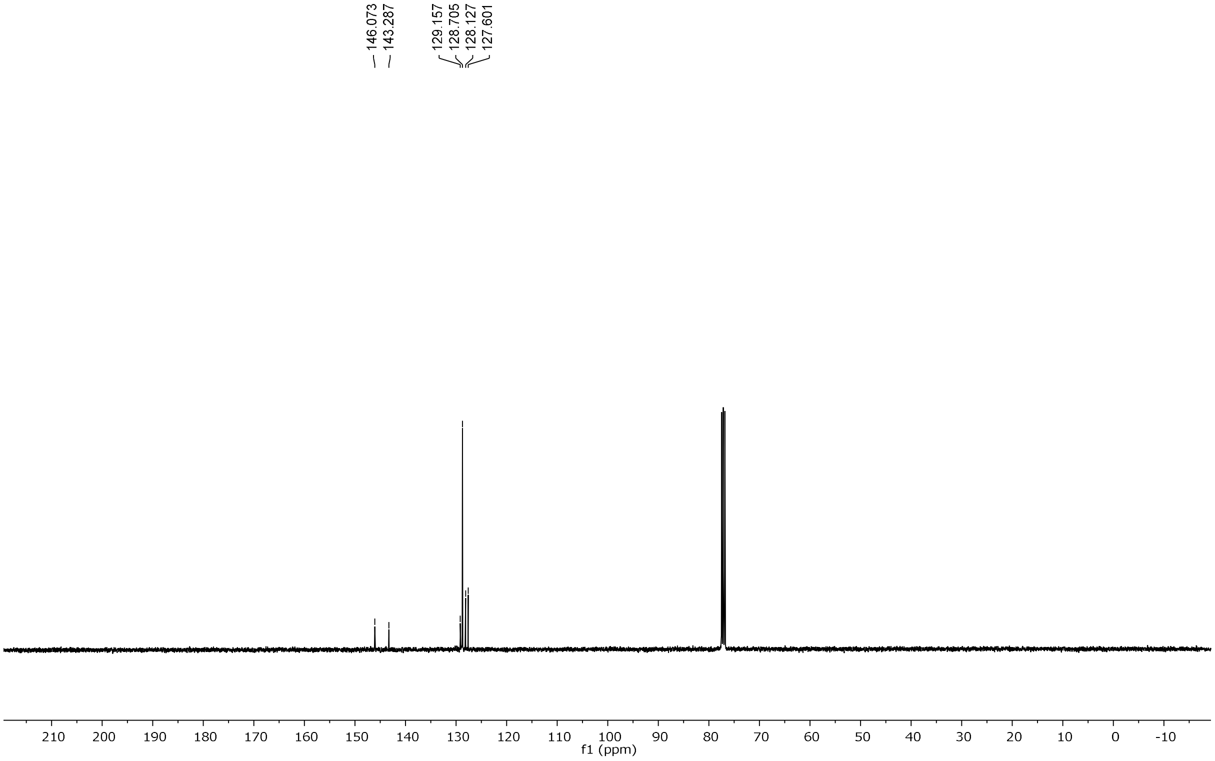

^{13}C NMR (101 MHz, CDCl3) of compound **1**

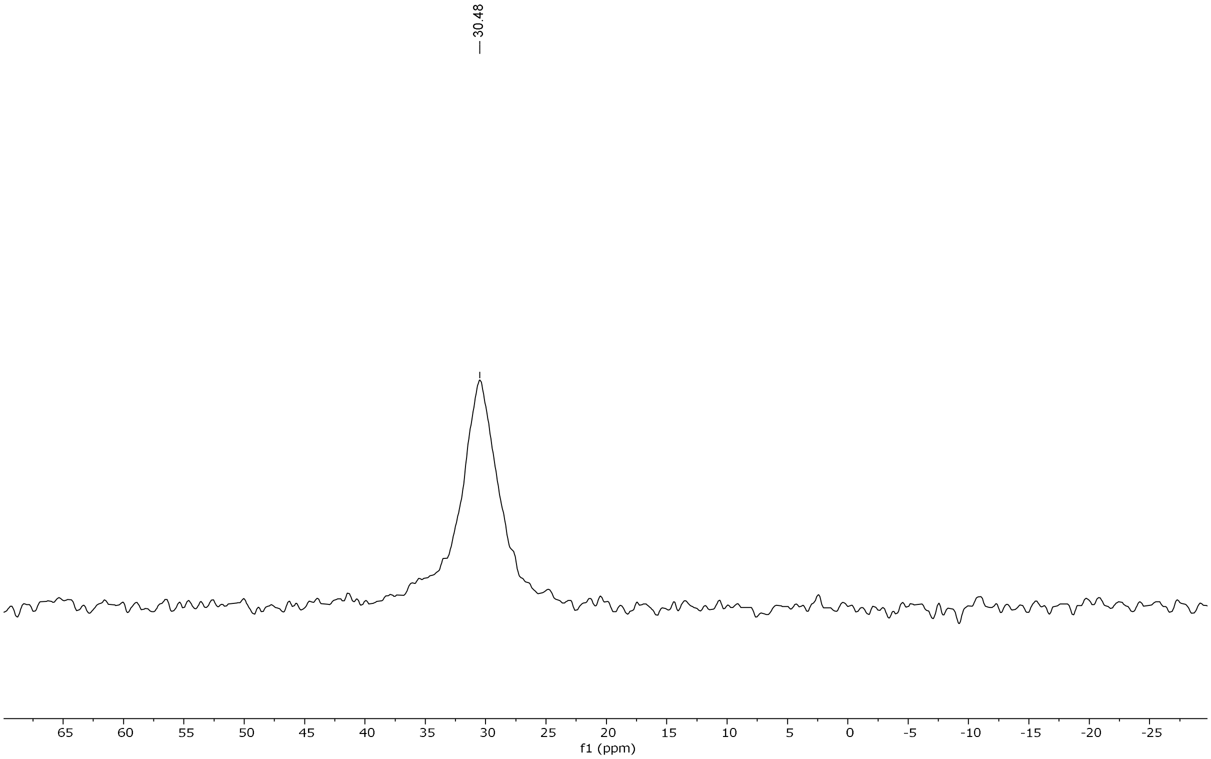

^{11}B NMR (160 MHz, $CDCl_3$) of compound **1**

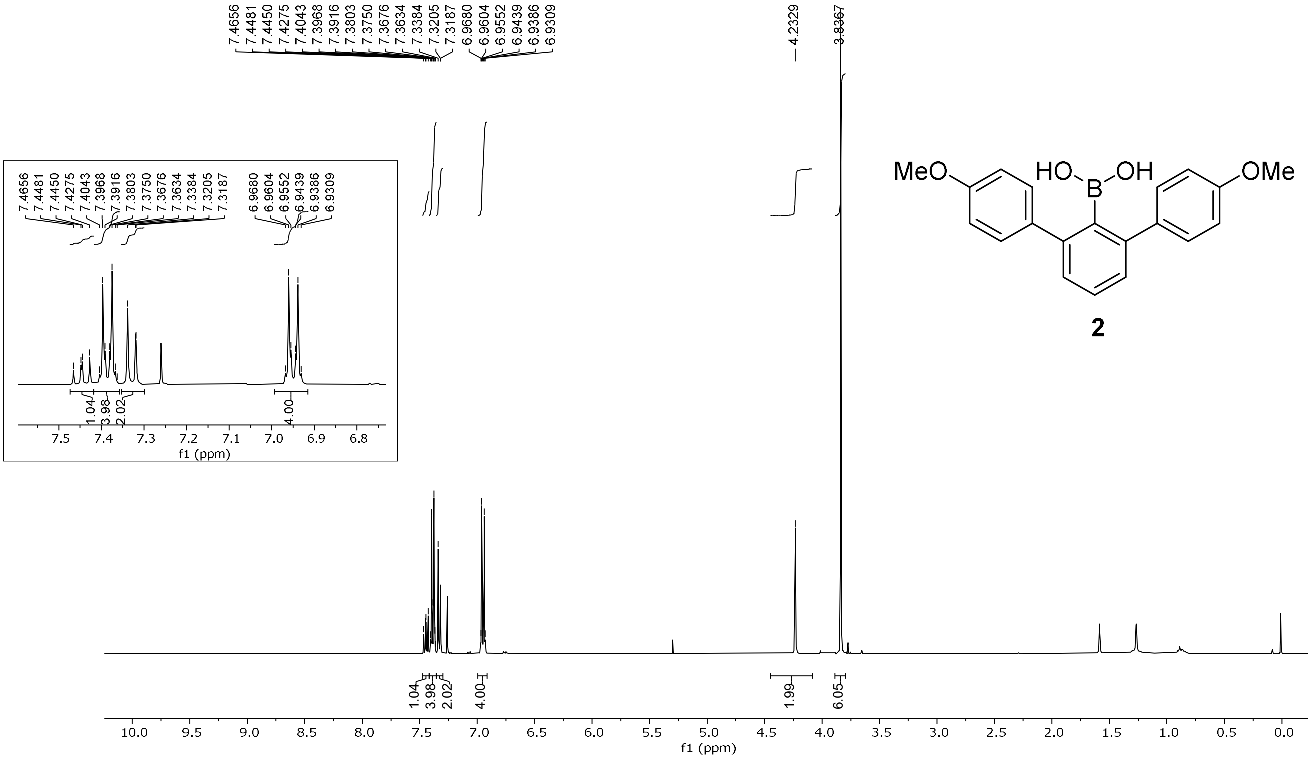

2

^{1}H NMR (400 MHz, $CDCl_3$) of compound **2**

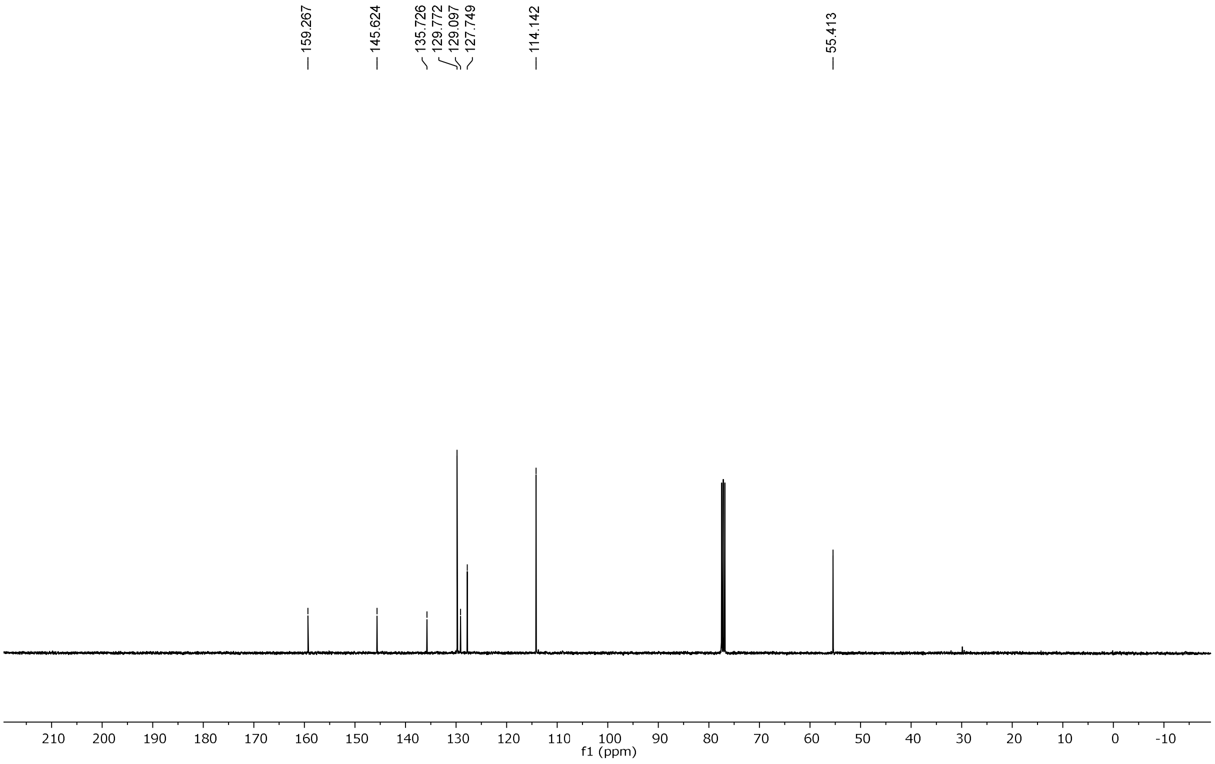

^{13}C NMR (101 MHz, $CDCl_3$) of compound **2**

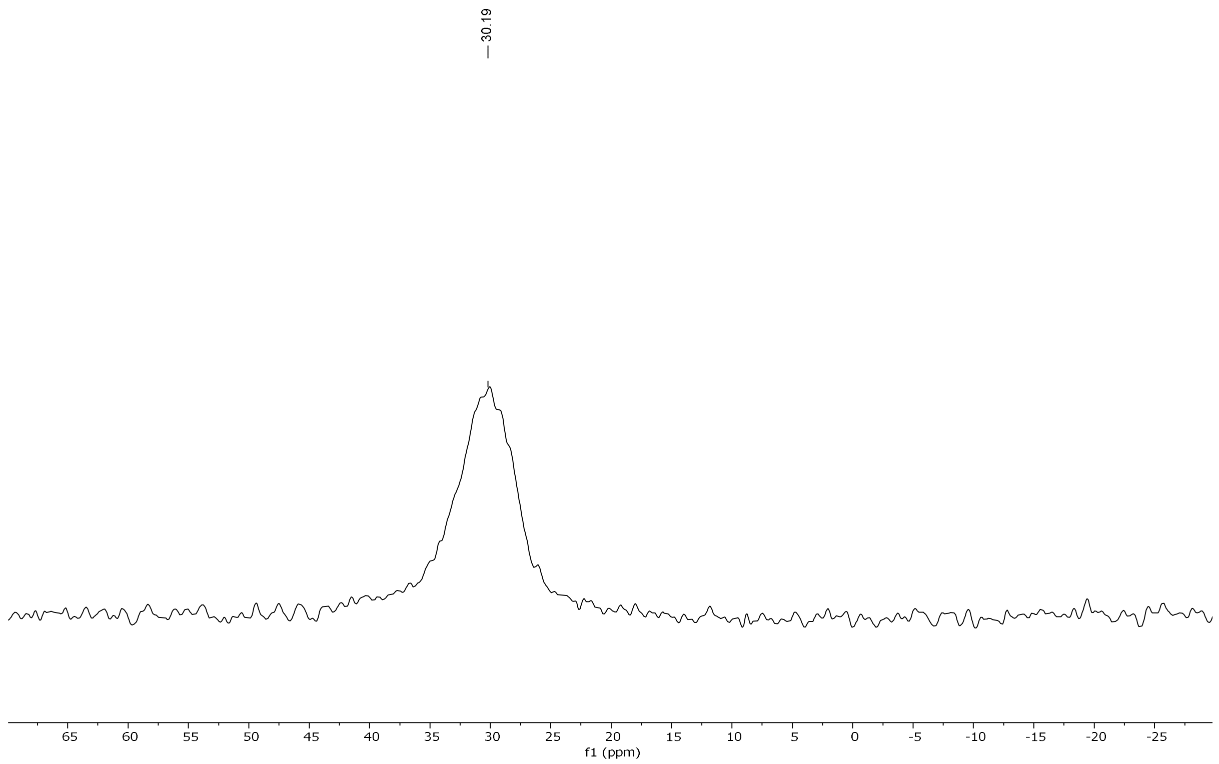

^{11}B NMR (160 MHz, $CDCl_3$) of compound **2**

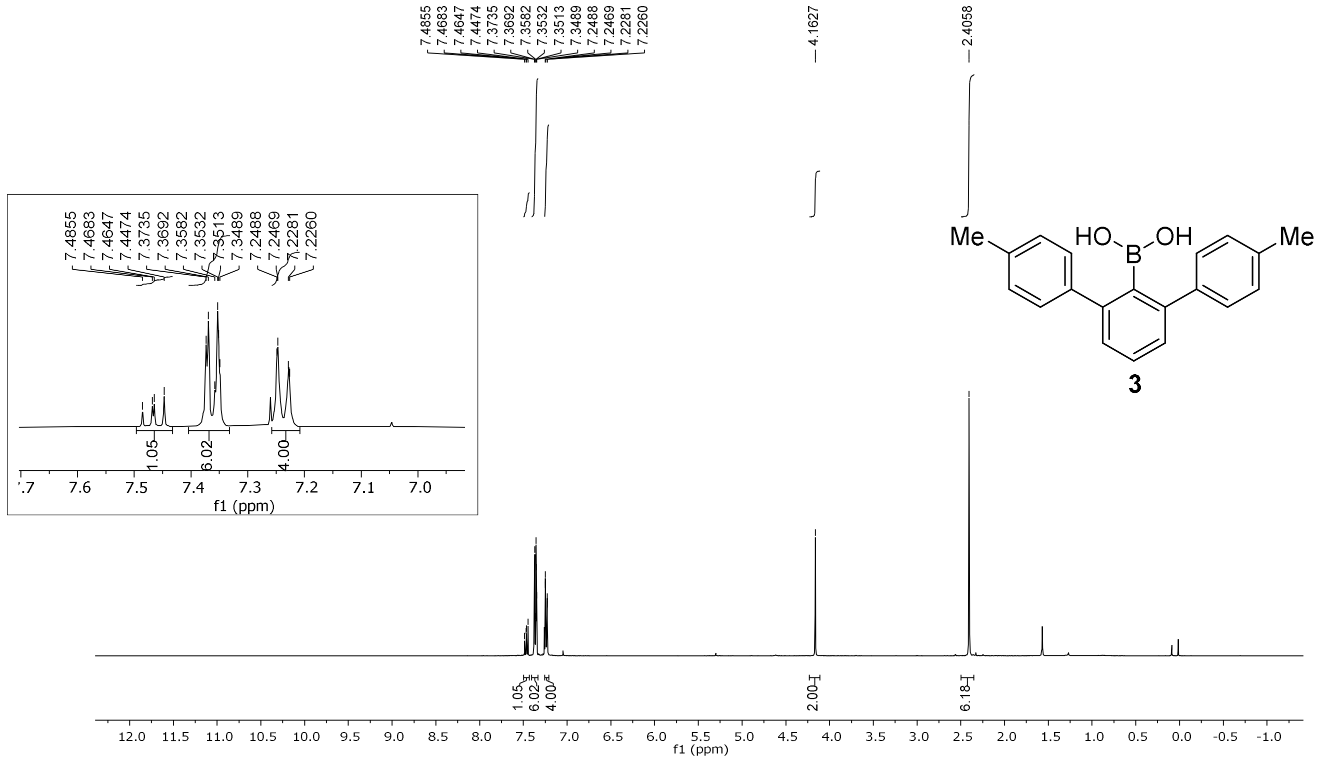

^{1}H NMR (400 MHz, $CDCl_3$) of compound **3**

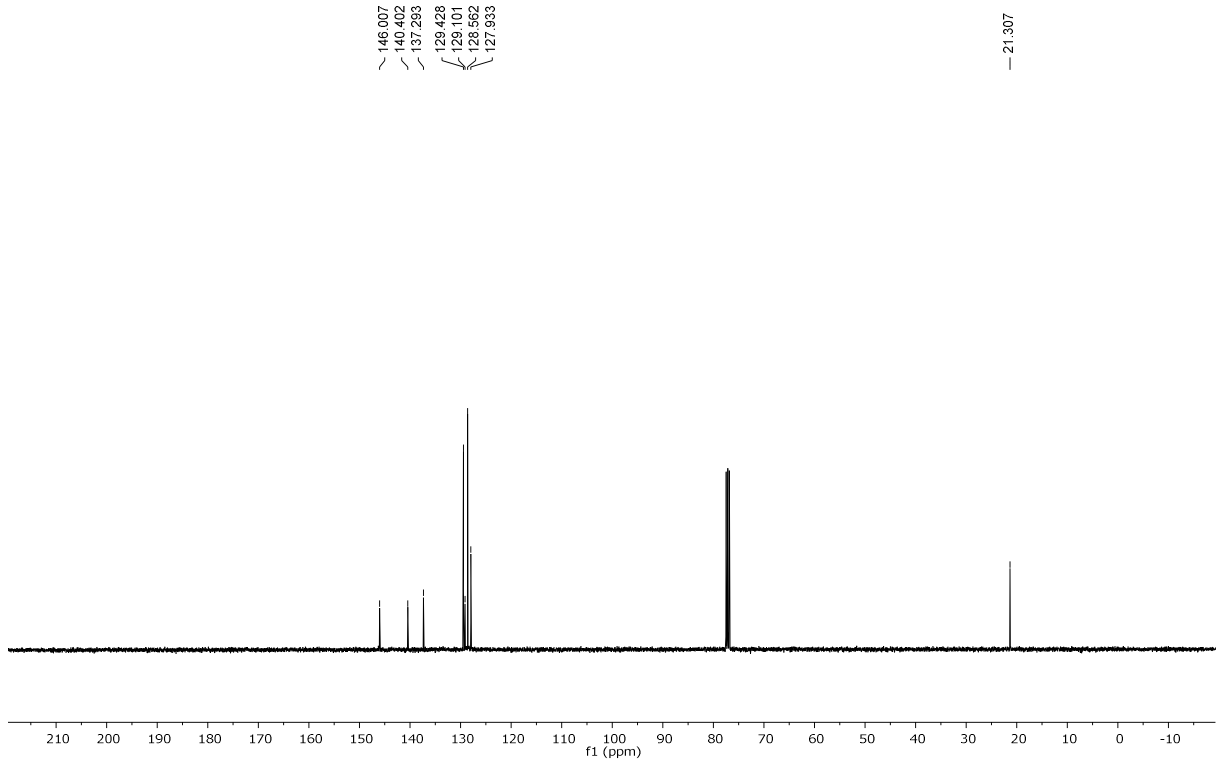

^{13}C NMR (101 MHz, $CDCl_3$) of compound **3**

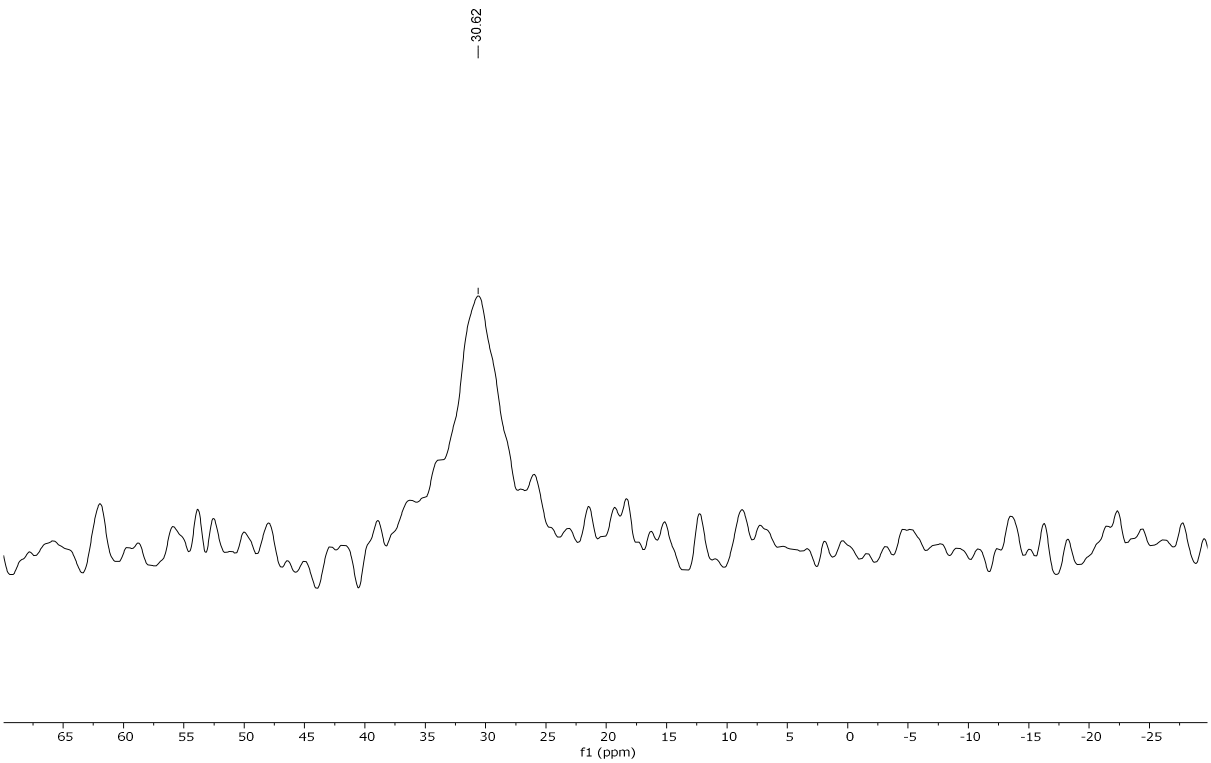

^{11}B NMR (160 MHz, $CDCl_3$) of compound **3**

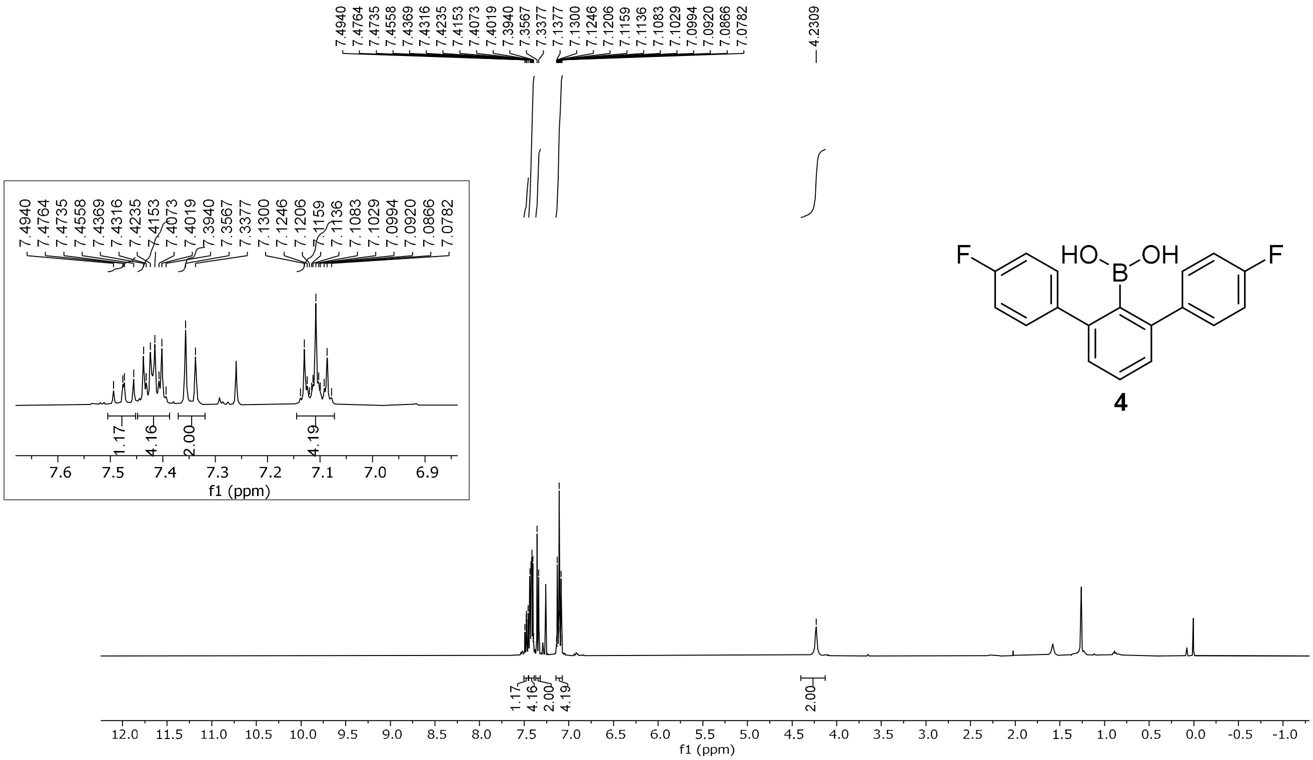

4

^{1}H NMR (400 MHz, CDCl$_3$) of compound **4**

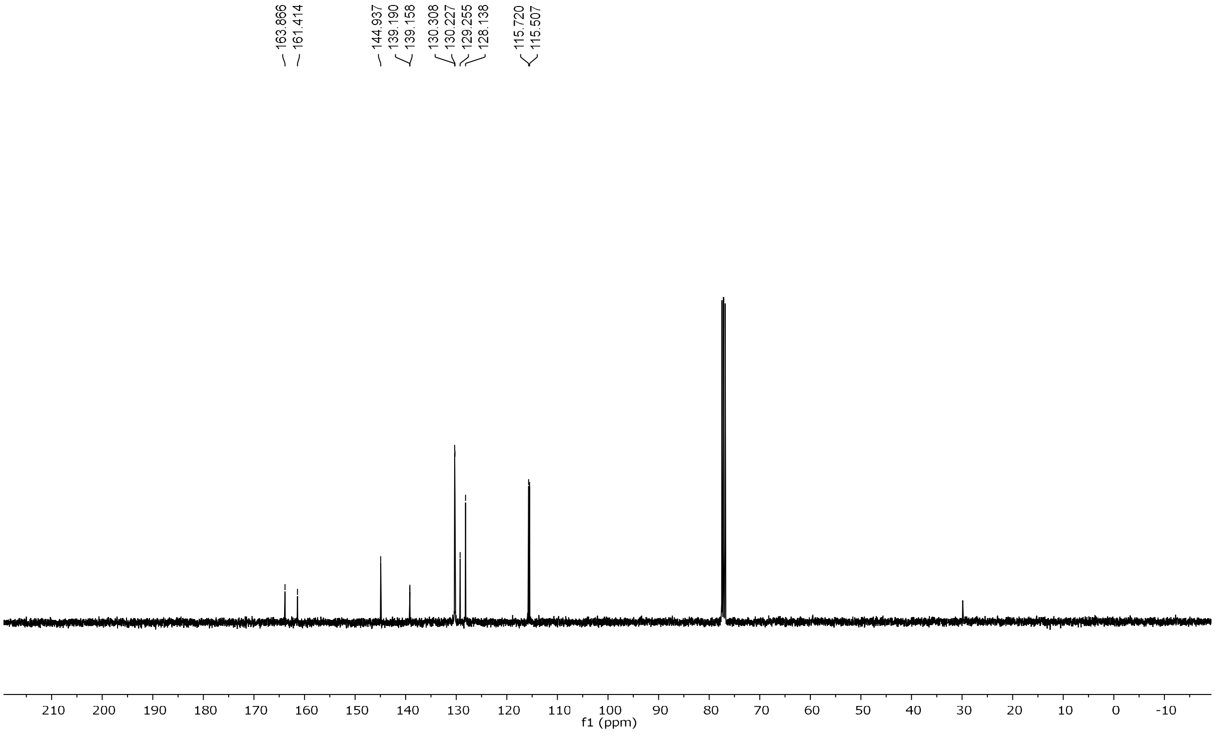

^{13}C NMR (101 MHz, CDCl$_3$) of compound **4**

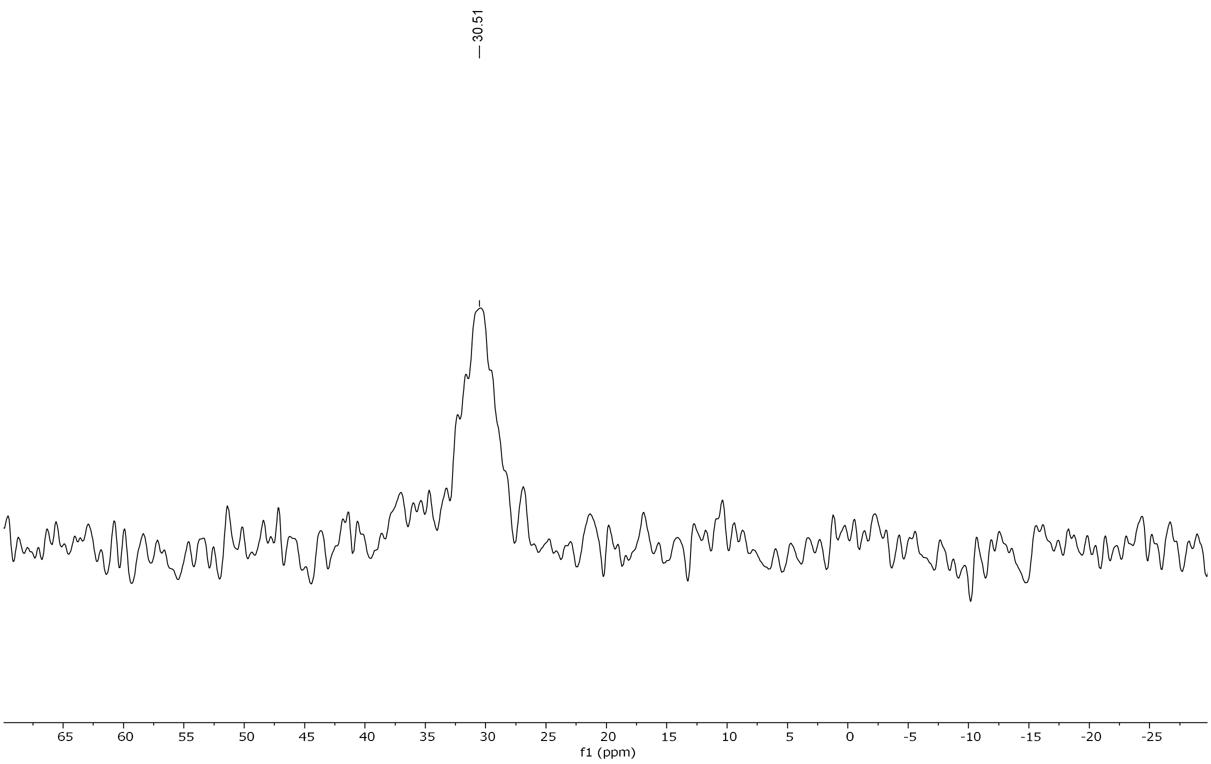

^{11}B NMR (160 MHz, $CDCl_3$) of compound **4**

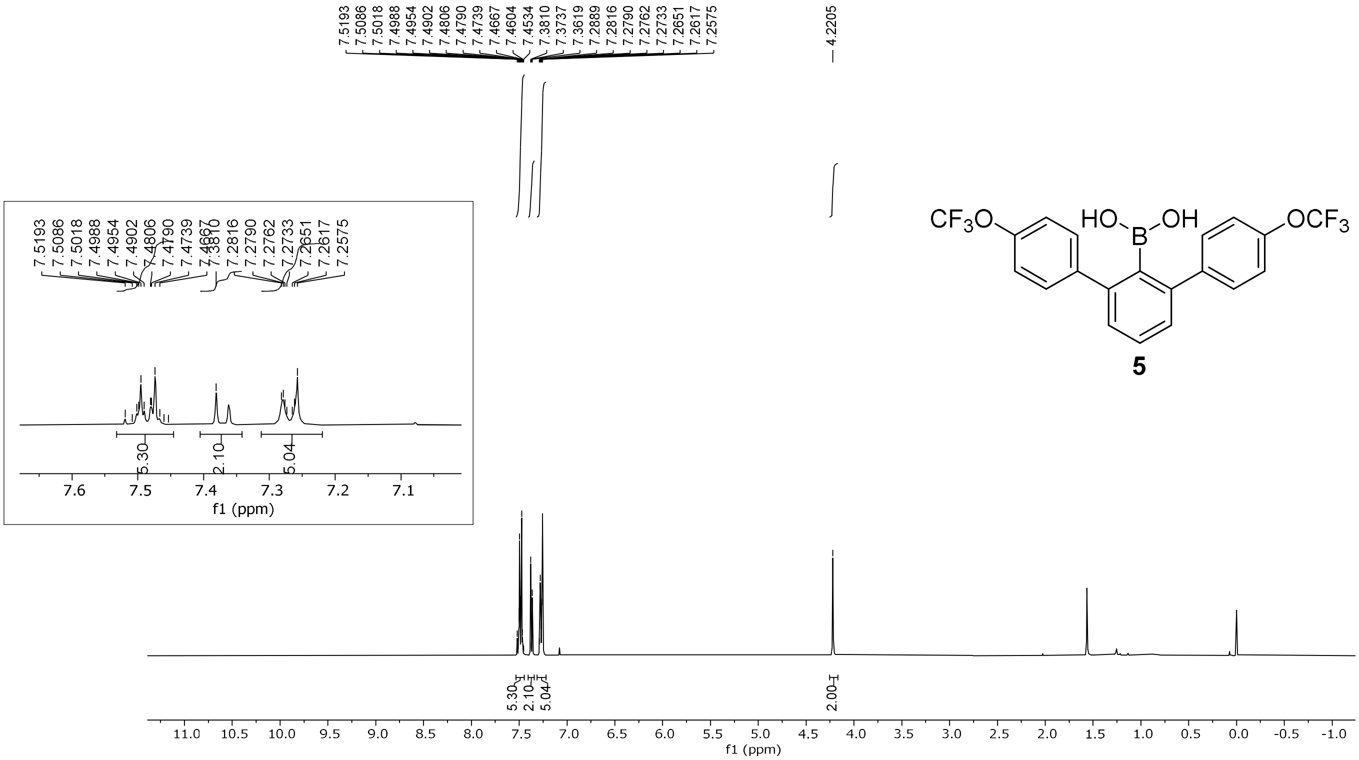

5

^{1}H NMR (400 MHz, $CDCl_3$) of compound **5**

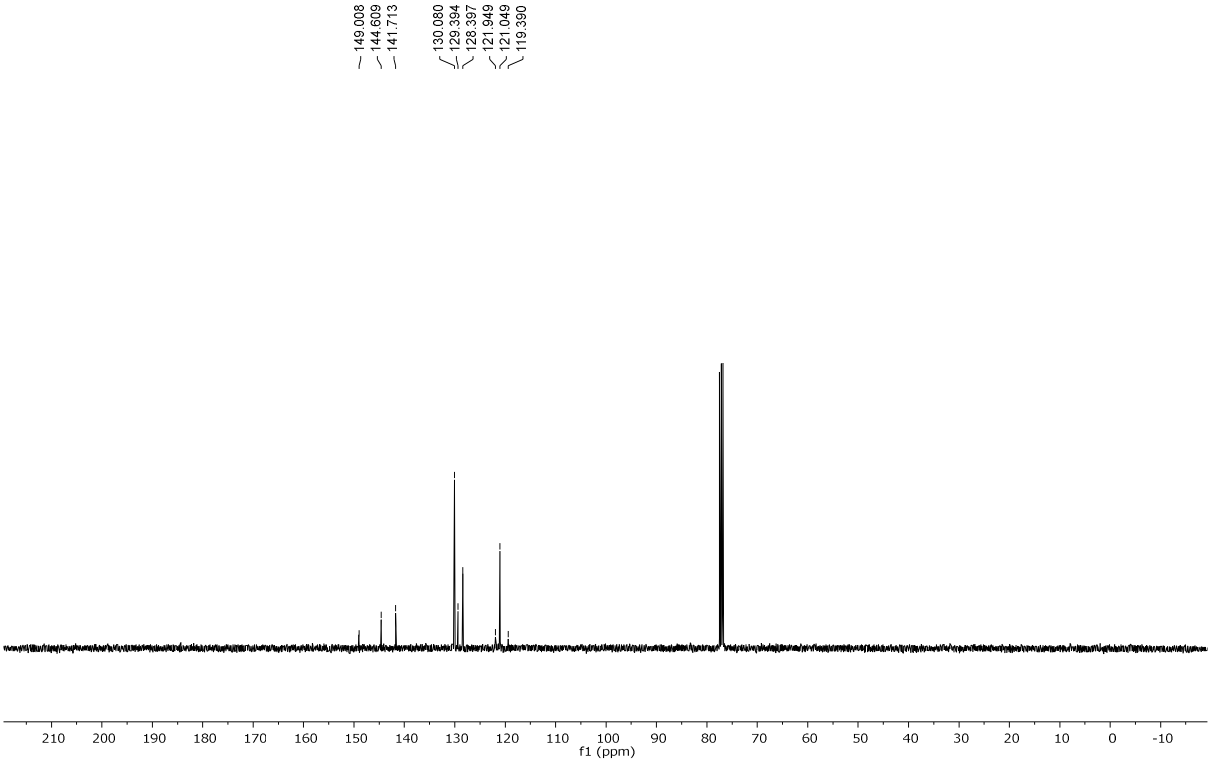

^{13}C NMR (101 MHz, $CDCl_3$) of compound **5**

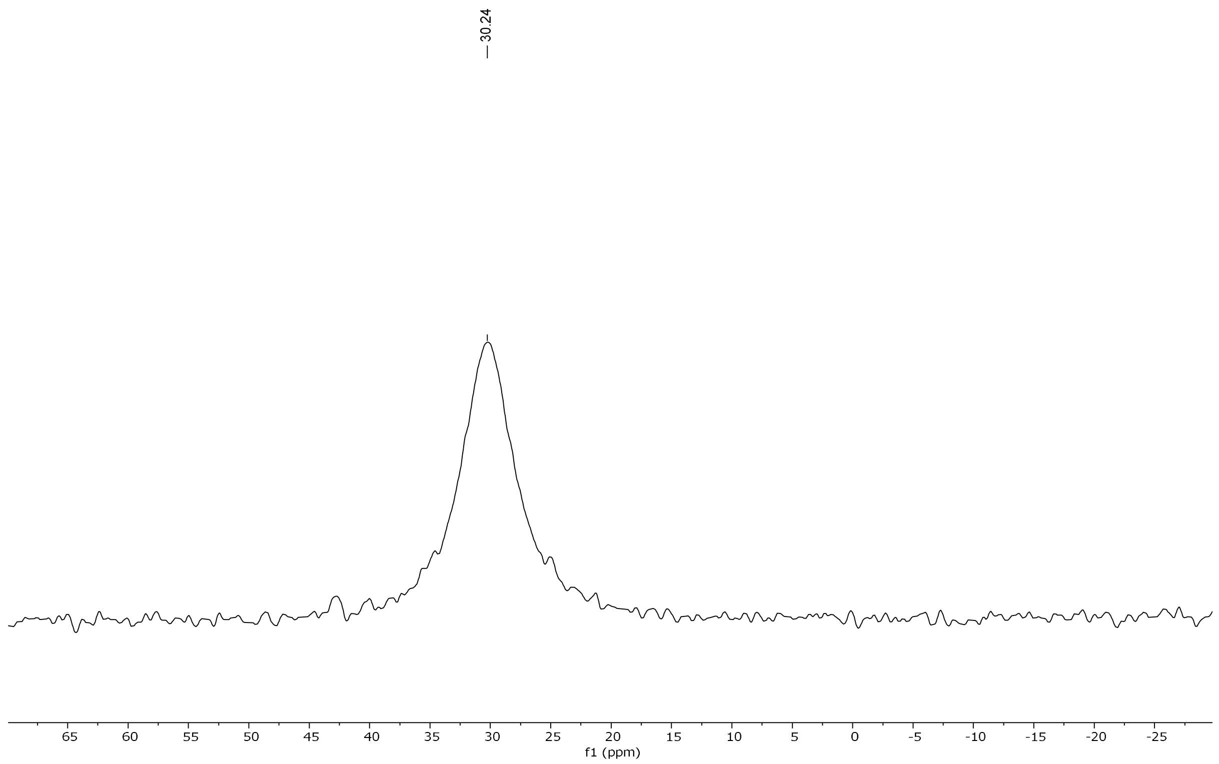

^{11}B NMR (160 MHz, $CDCl_3$) of compound **5**

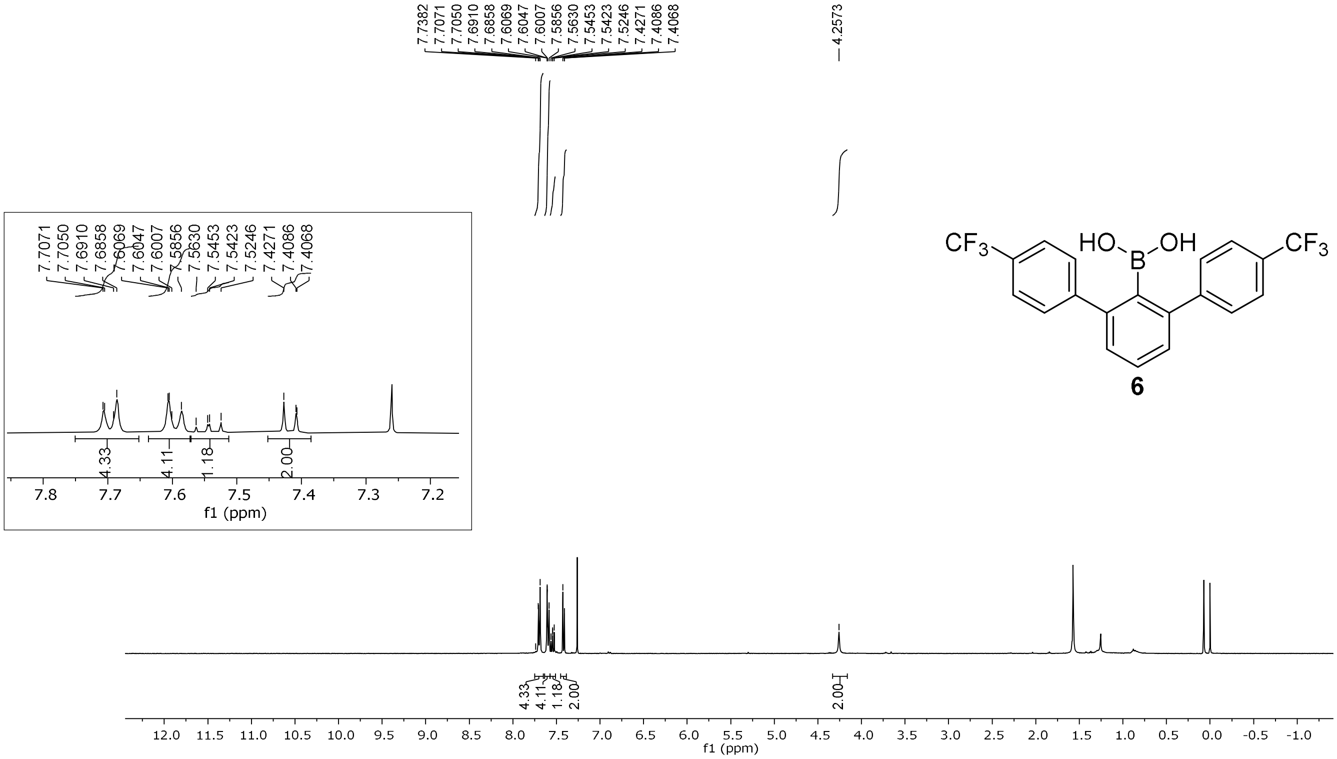

6

^{1}H NMR (400 MHz, CDCl$_3$) of compound **6**

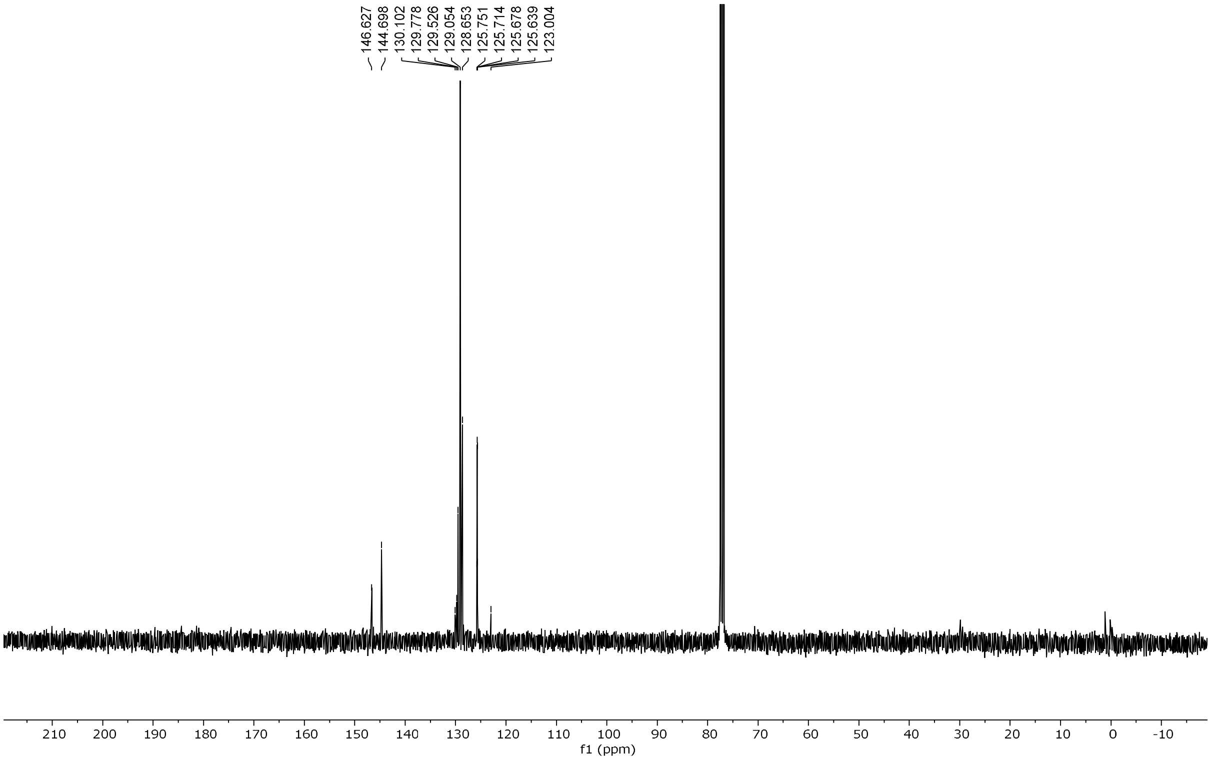

^{13}C NMR (101 MHz, CDCl$_3$) of compound **6**

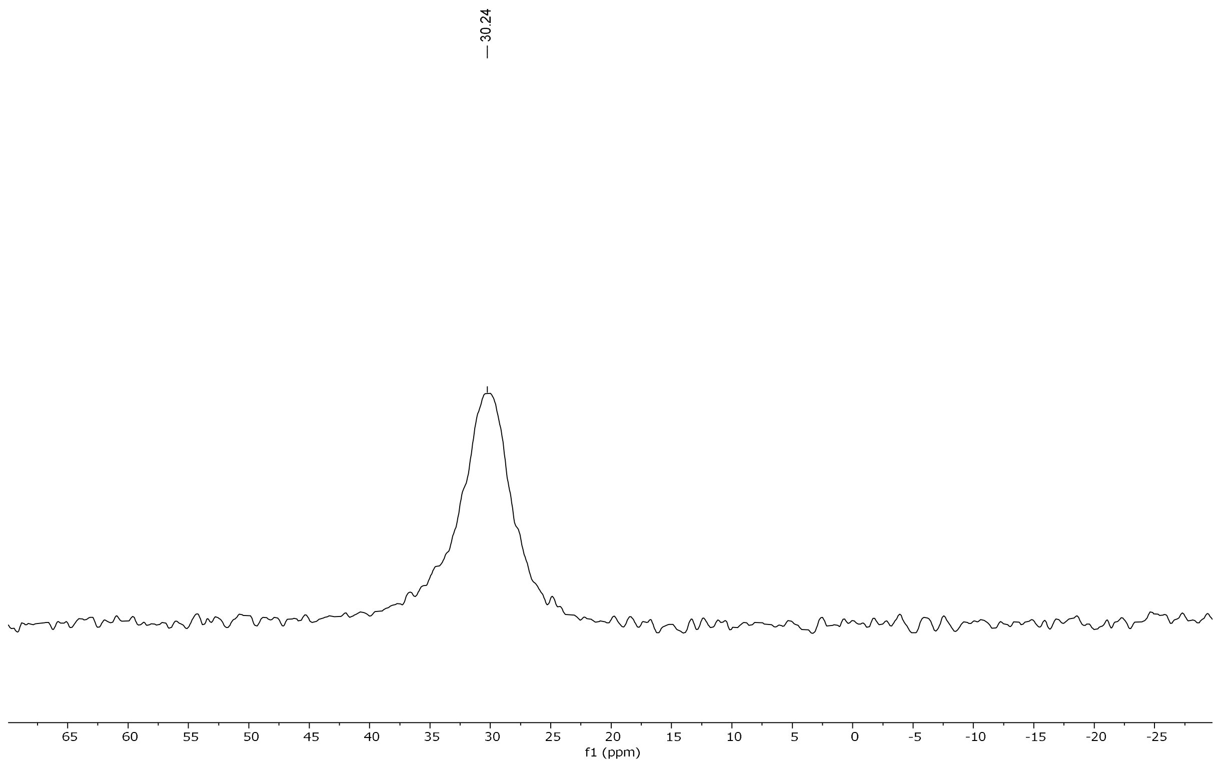

^{11}B NMR (160 MHz, $CDCl_3$) of compound **6**

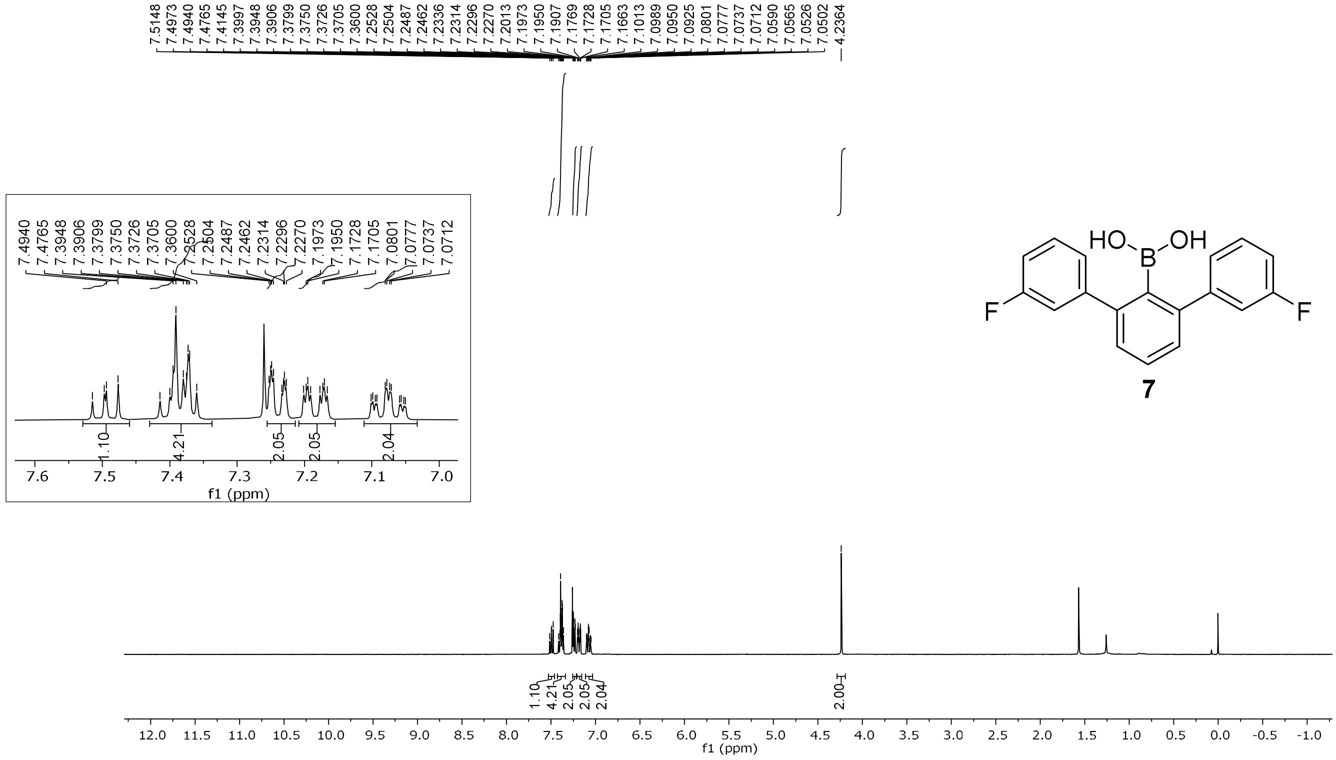

^{1}H NMR (400 MHz, $CDCl_3$) of compound **7**

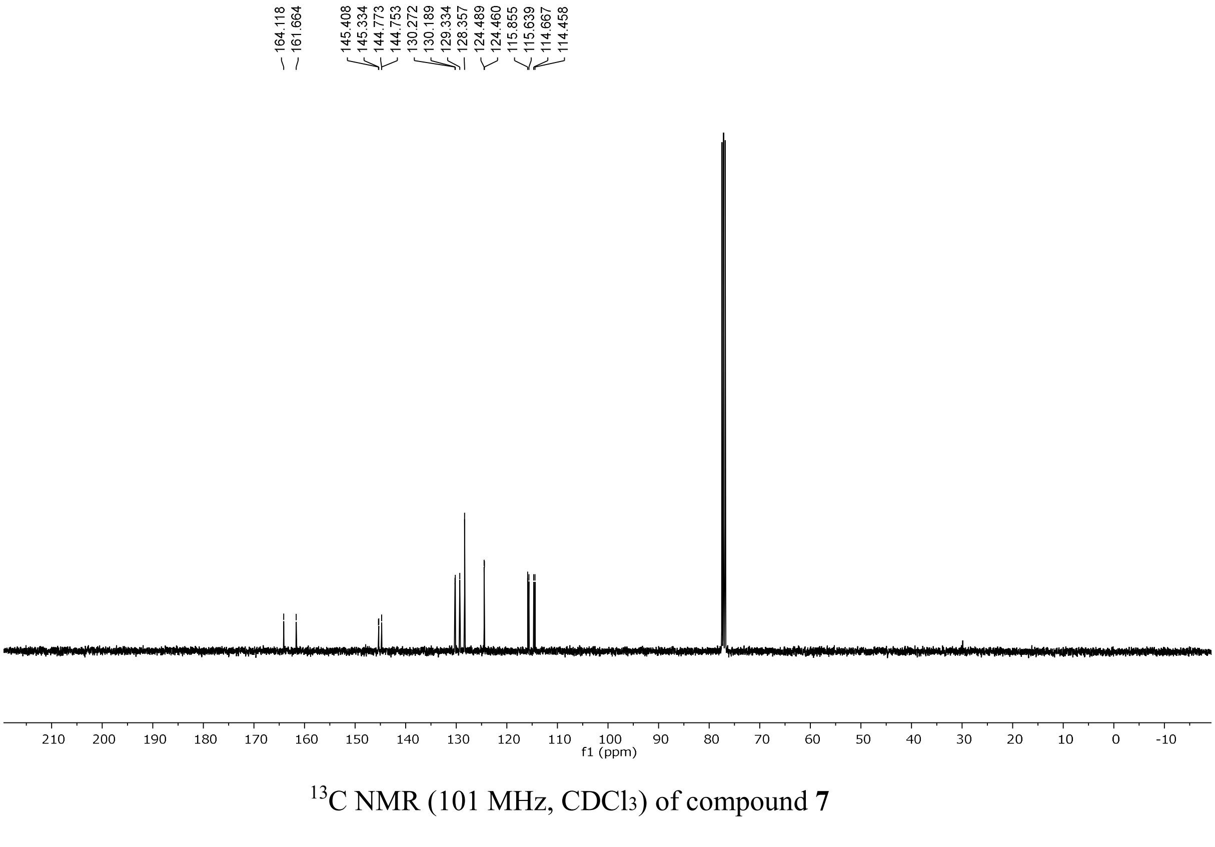

^{13}C NMR (101 MHz, $CDCl_3$) of compound **7**

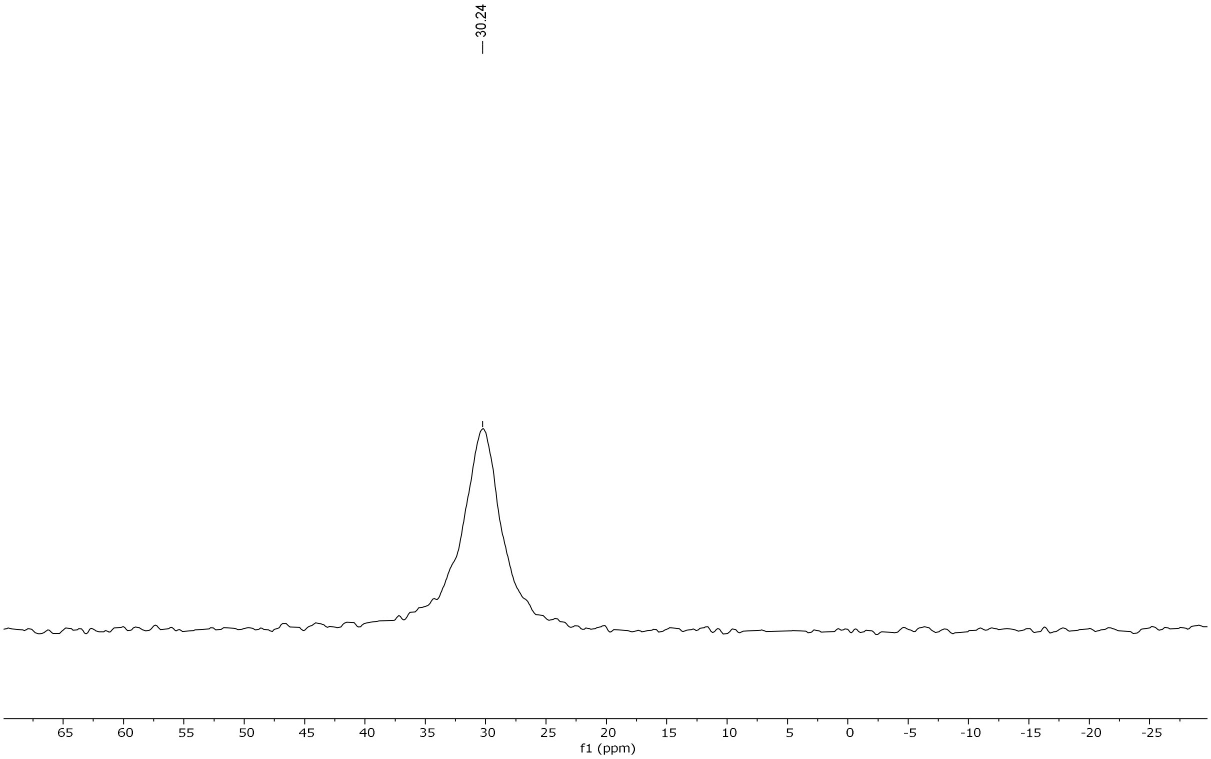

^{11}B NMR (160 MHz, $CDCl_3$) of compound **7**

2. pK_a measurements

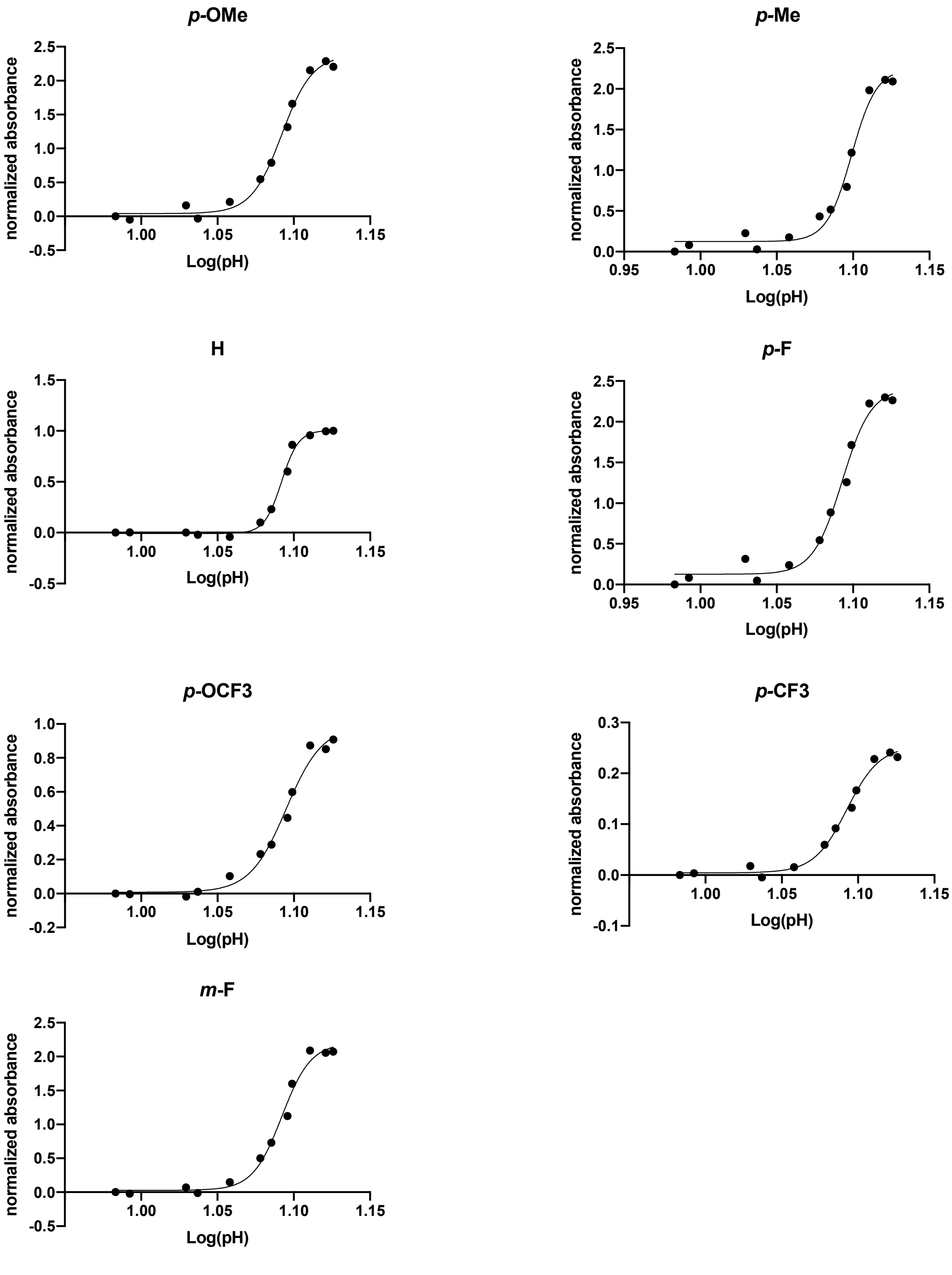

Figure S1. pK_a measurements of phenylboronic acids **1-7** using a 4-parameter fit.

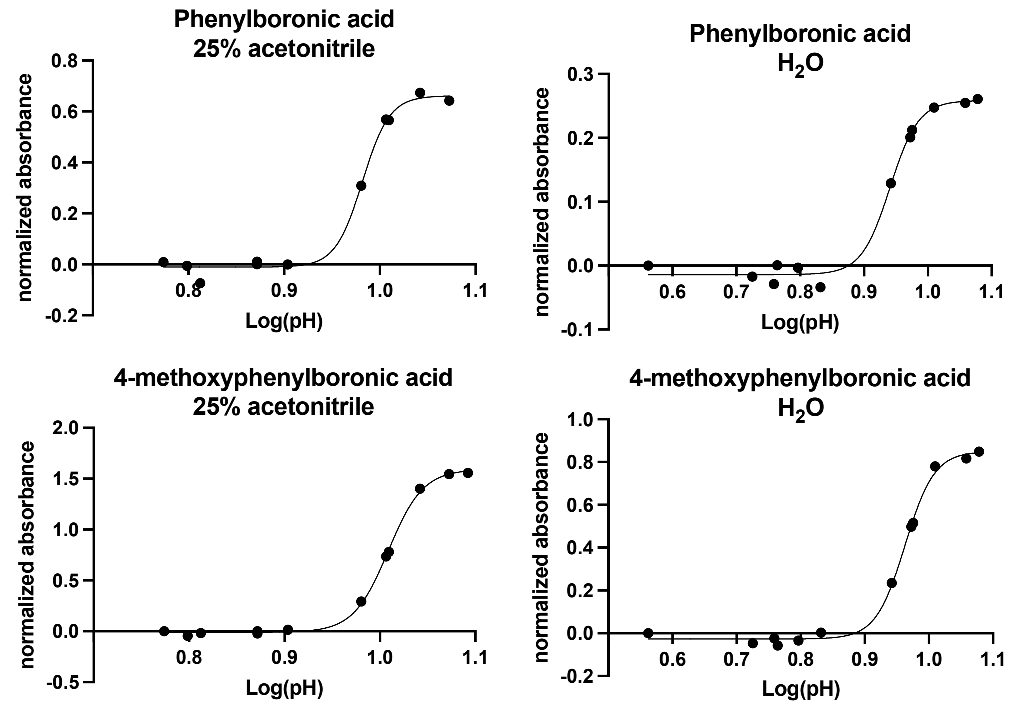

Figure S2. pK_a measurements of phenylboronic acid and 4-methoxyphenylboronic acid in 25% acetonitrile and in water. Data was fitted using a using a 4-parameter fit.

Table S1. pK_a values for control boronic acids.

**compd**	**pK_a determined in H_2O/acetonitrile = 3:1**	**pK_a determined in H_2O**
**phenylboronic acid**	9.61	8.68
**4-methoxyphenylboronic acid**	10.29	9.25

3. Single crystal X-ray crystallography

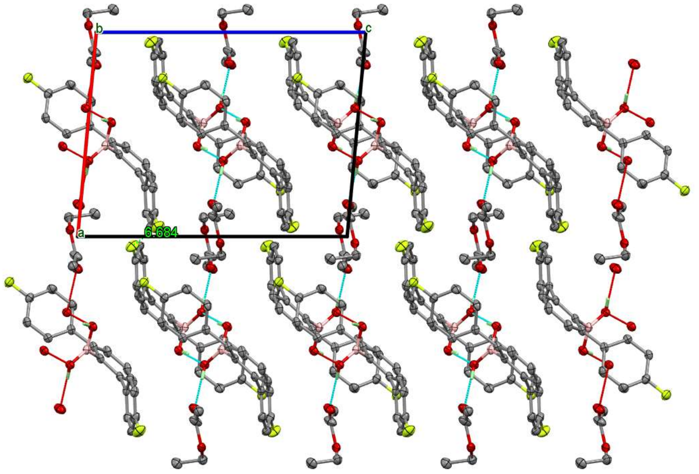

Figure S3. Packing with view down the b axis in the X-ray structure of **4**. Non-hydrogen atoms are drawn with 50% probability ellipsoids. Only the H atoms involved in H atom bonding (pale blue lines) are shown.

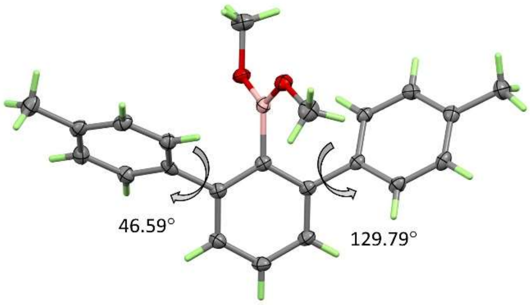

Figure S4. The X-ray structure of 2,6-diarylphenylboronic acid $\mathbf{3^{Me}}$. Showing the angles between the planes of the flanking rings relative to the central ring with rotation of both towards the reader. Non-hydrogen atoms are drawn with 50% probability ellipsoids.

4. Quantum Chemical Analyses

Table S2. Cartesian coordinates and absolute energy (in parentheses and in kcal mol^{-1}) of substituted 2,6-diaryl-phenylboronic acids computed at BLYP-D3BJ/TZ2P in water. Geometries confirmed to be minima by frequency analysis.

p-OMe (-6328.79)

1.C	0.000000	0.000000	3.577198
2.C	0.074303	-1.205309	2.878518
3.C	0.062907	-1.220651	1.473024
4.C	0.000000	0.000000	0.750020
5.C	-0.062907	1.220651	1.473024
6.C	-0.074303	1.205309	2.878518
7.H	0.110881	-2.146980	3.420434
8.H	1.825100	-3.275262	1.813124
9.H	1.671578	-1.022802	-1.043573
10.H	1.675418	2.151065	-0.423774
11.H	-0.110881	2.146980	3.420434
12.H	0.000000	0.000000	4.664738
13.C	0.079555	-2.535167	0.772890
14.C	-0.887310	-2.862722	-0.190006
15.C	-0.884006	-4.098212	-0.845525
16.C	0.108754	-5.040469	-0.538757
17.C	1.081766	-4.734028	0.428616
18.C	1.059958	-3.502039	1.074967
19.H	-1.675418	-2.151065	-0.423774
20.H	-1.655393	-4.313189	-1.576151
21.H	-0.505416	-7.641582	-2.457431
22.H	1.849323	-5.468963	0.657696
23.C	-0.079555	2.535167	0.772890
24.C	-1.059958	3.502039	1.074967
25.C	-1.081766	4.734028	0.428616
26.C	-0.108754	5.040469	-0.538757
27.C	0.884006	4.098212	-0.845525
28.C	0.887310	2.862722	-0.190006
29.H	-1.825100	3.275262	1.813124
30.H	-1.849323	5.468963	0.657696
31.H	0.505416	7.641582	-2.457431
32.H	1.655393	4.313189	-1.576151
33.B	0.000000	0.000000	-0.831393
34.O	0.998719	-0.580765	-1.588348
35.H	-1.671578	1.022802	-1.043573
36.O	-0.998719	0.580765	-1.588348
37.O	-0.210323	6.283920	-1.123032
38.C	0.771348	6.636005	-2.130276
39.H	1.781499	6.637064	-1.704873
40.H	0.724113	5.942628	-2.977804
41.O	0.210323	-6.283920	-1.123032
42.C	-0.771348	-6.636005	-2.130276
43.H	-1.781499	-6.637064	-1.704873
44.H	-0.724113	-5.942628	-2.977804

p-Me (-6053.58)

1.C	0.000000	0.000000	-3.364734
2.C	0.022174	1.207478	-2.665903
3.C	0.009699	1.221170	-1.260904
4.C	0.000000	0.000000	-0.537617

5.C	-0.009699	-1.221170	-1.260904
6.C	-0.022174	-1.207478	-2.665903
7.H	0.019696	2.150174	-3.207124
8.H	1.702523	3.334164	-1.566372
9.H	1.634492	1.081987	1.255672
10.H	1.793936	-2.078082	0.605070
11.H	-0.019696	-2.150174	-3.207124
12.H	0.000000	0.000000	-4.452200
13.C	-0.029659	2.533346	-0.556297
14.C	-1.026562	2.818904	0.393126
15.C	-1.062315	4.051568	1.047626
16.C	-0.103876	5.042966	0.781961
17.C	0.884499	4.760621	-0.175281
18.C	0.920878	3.530900	-0.836423
19.H	-1.793936	2.078082	0.605070
20.H	-1.851783	4.249501	1.770161
21.H	-1.136056	6.632566	1.829572
22.H	1.637601	5.512287	-0.404547
23.C	0.029659	-2.533346	-0.556297
24.C	-0.920878	-3.530900	-0.836423
25.C	-0.884499	-4.760621	-0.175281
26.C	0.103876	-5.042966	0.781961
27.C	1.062315	-4.051568	1.047626
28.C	1.026562	-2.818904	0.393126
29.H	-1.702523	-3.334164	-1.566372
30.H	-1.637601	-5.512287	-0.404547
31.H	-0.496686	-6.308787	2.426723
32.H	1.851783	-4.249501	1.770161
33.B	0.000000	0.000000	1.044190
34.O	0.976369	0.618004	1.800123
35.H	-1.634492	-1.081987	1.255672
36.O	-0.976369	-0.618004	1.800123
37.C	0.120799	-6.363292	1.519529
38.H	-0.280771	-7.171199	0.898468
39.H	1.136056	-6.632566	1.829572
40.C	-0.120799	6.363292	1.519529
41.H	0.496686	6.308787	2.426723
42.H	0.280771	7.171199	0.898468

H (-5316.55)

1.C	0.000000	0.000000	-3.065761
2.C	0.007302	1.207514	-2.366787
3.C	-0.005220	1.220338	-0.962074
4.C	0.000000	0.000000	-0.238226
5.C	0.005220	-1.220338	-0.962074
6.C	-0.007302	-1.207514	-2.366787
7.H	-0.006225	2.150198	-2.907775
8.H	1.674887	3.336881	-1.260631
9.H	1.639203	1.076249	1.555448
10.H	1.822100	-2.049989	0.895452
11.H	0.006225	-2.150198	-2.907775
12.H	0.000000	0.000000	-4.153121
13.C	-0.059612	2.532693	-0.257493
14.C	-1.065058	2.802459	0.688742
15.C	-1.114797	4.033652	1.347513
16.C	-0.158769	5.017837	1.071132
17.C	0.842046	4.764229	0.125852
18.C	0.888699	3.533790	-0.535959
19.H	-1.822100	2.049989	0.895452
20.H	-1.902826	4.226119	2.071581

21.H	-0.195159	5.975073	1.585426
22.H	1.590052	5.522576	-0.092896
23.C	0.059612	-2.532693	-0.257493
24.C	-0.888699	-3.533790	-0.535959
25.C	-0.842046	-4.764229	0.125852
26.C	0.158769	-5.017837	1.071132
27.C	1.114797	-4.033652	1.347513
28.C	1.065058	-2.802459	0.688742
29.H	-1.674887	-3.336881	-1.260631
30.H	-1.590052	-5.522576	-0.092896
31.H	0.195159	-5.975073	1.585426
32.H	1.902826	-4.226119	2.071581
33.B	0.000000	0.000000	1.343934
34.O	0.975855	0.618859	2.099056
35.H	-1.639203	-1.076249	1.555448
36.O	-0.975855	-0.618859	2.099056

p-F (-5329.11)

1.C	0.000000	0.000000	-3.073556
2.C	0.018818	1.207376	-2.374716
3.C	0.007160	1.220073	-0.970099
4.C	0.000000	0.000000	-0.246147
5.C	-0.007160	-1.220073	-0.970099
6.C	-0.018818	-1.207376	-2.374716
7.H	0.013556	2.149965	-2.915946
8.H	1.702852	3.327118	-1.277520
9.H	1.664057	1.038243	1.547813
10.H	1.794044	-2.067462	0.900536
11.H	-0.013556	-2.149965	-2.915946
12.H	0.000000	0.000000	-4.160858
13.C	-0.032610	2.532371	-0.264818
14.C	-1.029871	2.809797	0.687671
15.C	-1.073136	4.037354	1.353103
16.C	-0.102026	4.982287	1.047098
17.C	0.897256	4.759120	0.108149
18.C	0.921704	3.525885	-0.549079
19.H	-1.794044	2.067462	0.900536
20.H	-1.844715	4.258915	2.084229
21.F	-0.133497	6.198955	1.702640
22.H	1.638061	5.525921	-0.097649
23.C	0.032610	-2.532371	-0.264818
24.C	-0.921704	-3.525885	-0.549079
25.C	-0.897256	-4.759120	0.108149
26.C	0.102026	-4.982287	1.047098
27.C	1.073136	-4.037354	1.353103
28.C	1.029871	-2.809797	0.687671
29.H	-1.702852	-3.327118	-1.277520
30.H	-1.638061	-5.525921	-0.097649
31.F	0.133497	-6.198955	1.702640
32.H	1.844715	-4.258915	2.084229
33.B	0.000000	0.000000	1.336072
34.O	0.988061	0.599033	2.090833
35.H	-1.664057	-1.038243	1.547813
36.O	-0.988061	-0.599033	2.090833

p-OCF_3 (-6391.41)

1.C	0.000000	0.000000	-3.339995
2.C	0.103991	1.203559	-2.641460
3.C	0.092616	1.215743	-1.237280
4.C	0.000000	0.000000	-0.513422

5.C	-0.092616	-1.215743	-1.237280
6.C	-0.103991	-1.203559	-2.641460
7.H	0.166697	2.144031	-3.182898
8.H	1.949774	3.187468	-1.518411
9.H	1.760213	0.867031	1.281064
10.H	1.640591	-2.176615	0.637871
11.H	-0.166697	-2.144031	-3.182898
12.H	0.000000	0.000000	-4.427203
13.C	0.151986	2.521423	-0.521791
14.C	-0.817679	2.857477	0.439986
15.C	-0.748886	4.062611	1.140659
16.C	0.298596	4.933862	0.861093
17.C	1.271365	4.644237	-0.090340
18.C	1.187815	3.435259	-0.784925
19.H	-1.640591	2.176615	0.637871
20.H	-1.492041	4.320236	1.888361
21.F	0.042231	8.253995	2.031708
22.H	2.082809	5.341444	-0.273323
23.C	-0.151986	-2.521423	-0.521791
24.C	-1.187815	-3.435259	-0.784925
25.C	-1.271365	-4.644237	-0.090340
26.C	-0.298596	-4.933862	0.861093
27.C	0.748886	-4.062611	1.140659
28.C	0.817679	-2.857477	0.439986
29.H	-1.949774	-3.187468	-1.518411
30.H	-2.082809	-5.341444	-0.273323
31.F	-0.042231	-8.253995	2.031708
32.H	1.492041	-4.320236	1.888361
33.B	0.000000	0.000000	1.069659
34.O	1.041177	0.501443	1.823249
35.H	-1.760213	-0.867031	1.281064
36.O	-1.041177	-0.501443	1.823249
37.O	-0.424627	-6.132732	1.636586
38.C	0.188924	-7.243220	1.159717
39.F	-0.274221	-7.631357	-0.072520
40.F	1.547317	-7.098752	1.028135
41.O	0.424627	6.132732	1.636586
42.C	-0.188924	7.243220	1.159717
43.F	0.274221	7.631357	-0.072520
44.F	-1.547317	7.098752	1.028135

p-CF_3 (-6108.77)

1.C	0.000000	0.000000	3.377498
2.C	0.037176	-1.206593	2.678544
3.C	0.023025	-1.218164	1.274101
4.C	0.000000	0.000000	0.548533
5.C	-0.023025	1.218164	1.274101
6.C	-0.037176	1.206593	2.678544
7.H	0.046941	-2.149162	3.219392
8.H	1.734741	-3.308895	1.589618
9.H	1.703351	-0.975507	-1.246076
10.H	1.763873	2.080360	-0.600789
11.H	-0.046941	2.149162	3.219392
12.H	0.000000	0.000000	4.464562
13.C	-0.001551	-2.529886	0.569779
14.C	-0.992983	-2.815207	-0.387299
15.C	-1.016127	-4.039727	-1.048904
16.C	-0.042088	-5.005452	-0.758690
17.C	0.946028	-4.742636	0.196095
18.C	0.959362	-3.513991	0.857042

19.H	-1.763873	-2.080360	-0.600789
20.H	-1.792313	-4.245038	-1.780055
21.F	-1.318290	-6.877040	-1.534840
22.H	1.702723	-5.486554	0.421021
23.C	0.001551	2.529886	0.569779
24.C	-0.959362	3.513991	0.857042
25.C	-0.946028	4.742636	0.196095
26.C	0.042088	5.005452	-0.758690
27.C	1.016127	4.039727	-1.048904
28.C	0.992983	2.815207	-0.387299
29.H	-1.734741	3.308895	1.589618
30.H	-1.702723	5.486554	0.421021
31.F	-0.296193	6.146037	-2.836707
32.H	1.792313	4.245038	-1.780055
33.B	0.000000	0.000000	-1.034502
34.O	1.005554	-0.569594	-1.787266
35.H	-1.703351	0.975507	-1.246076
36.O	-1.005554	0.569594	-1.787266
37.C	0.064090	6.306721	-1.509524
38.F	-0.784480	7.246791	-0.993485
39.F	1.318290	6.877040	-1.534840
40.C	-0.064090	-6.306721	-1.509524
41.F	0.296193	-6.146037	-2.836707
42.F	0.784480	-7.246791	-0.993485

m-F (-5329.27)

1.C	0.000000	0.000000	-3.064053
2.C	-0.117671	1.201502	-2.365030
3.C	-0.132028	1.211499	-0.960624
4.C	0.000000	0.000000	-0.235690
5.C	0.132028	-1.211499	-0.960624
6.C	0.117671	-1.201502	-2.365030
7.H	-0.228640	2.137716	-2.906011
8.H	1.320100	3.511294	-1.272018
9.H	1.565806	1.182310	1.558065
10.H	2.015266	-1.847412	0.913291
11.H	0.228640	-2.137716	-2.906011
12.H	0.000000	0.000000	-4.151223
13.C	-0.322624	2.509824	-0.254137
14.C	-1.342707	2.673472	0.700652
15.C	-1.515919	3.893092	1.359850
16.C	-0.677427	4.978492	1.081365
17.C	0.316884	4.795587	0.128311
18.C	0.516275	3.600040	-0.547558
19.H	-2.015266	1.847412	0.913291
20.H	-2.311403	4.005685	2.091376
21.H	-0.790906	5.935157	1.582197
22.F	1.157713	5.855417	-0.158496
23.C	0.322624	-2.509824	-0.254137
24.C	-0.516275	-3.600040	-0.547558
25.C	-0.316884	-4.795587	0.128311
26.C	0.677427	-4.978492	1.081365
27.C	1.515919	-3.893092	1.359850
28.C	1.342707	-2.673472	0.700652
29.H	-1.320100	-3.511294	-1.272018
30.F	-1.157713	-5.855417	-0.158496
31.H	0.790906	-5.935157	1.582197
32.H	2.311403	-4.005685	2.091376
33.B	0.000000	0.000000	1.347170
34.O	0.928624	0.688183	2.100665

35.H	-1.565806	-1.182310	1.558065
36.O	-0.928624	-0.688183	2.100665

Table S3. Cartesian coordinates and absolute energy (in parentheses and in kcal mol^{-1}) of anionic substituted 2,6-diaryl-phenylboronates computed at BLYP-D3BJ/TZ2P in water. Geometries confirmed to be minima by frequency analysis.

p-OMe (-6644.29)

1.C	0.125330	-1.015900	-0.878717
2.C	1.264198	-0.319459	-0.478923
3.C	1.175615	1.000567	0.003708
4.C	-0.073547	1.688203	0.072572
5.C	-1.226589	0.924776	-0.281382
6.C	-1.116386	-0.394928	-0.761517
7.H	2.238438	-0.802171	-0.521609
8.H	1.886564	1.888289	2.505924
9.H	-2.112582	3.603745	-0.090344
10.H	-2.550281	1.799175	1.960564
11.H	-2.021169	-0.938135	-1.026660
12.H	0.201628	-2.035636	-1.249842
13.C	2.472753	1.606013	0.444830
14.C	3.547510	1.738928	-0.449581
15.C	4.783382	2.260460	-0.049849
16.C	4.969686	2.651134	1.284413
17.C	3.915067	2.507487	2.201113
18.C	2.689666	1.993492	1.783179
19.H	3.412431	1.447490	-1.488376
20.H	5.578818	2.359447	-0.779966
21.H	8.059855	3.769342	1.464167
22.H	4.070436	2.800064	3.236607
23.C	-2.626464	1.443418	-0.163776
24.C	-3.474621	1.485758	-1.290403
25.C	-4.791917	1.923660	-1.193209
26.C	-5.311395	2.325244	0.049623
27.C	-4.495659	2.273110	1.188311
28.C	-3.170167	1.834398	1.070039
29.H	-3.084452	1.187956	-2.260515
30.H	-5.430639	1.966905	-2.072030
31.H	-8.213332	3.486722	1.079548
32.H	-4.871944	2.564023	2.162588
33.O	-1.266465	4.033368	-0.289941
34.O	1.021925	4.102351	-0.037322
35.H	1.831797	3.708467	0.322070
36.H	-0.343807	2.638834	2.364813
37.O	-6.624235	2.749106	0.047108
38.C	-7.189186	3.188692	1.306805
39.H	-7.192202	2.372372	2.038449
40.H	-6.631477	4.044020	1.705693
41.O	6.140993	3.178883	1.787194
42.C	7.250444	3.344195	0.869813
43.H	7.560203	2.378013	0.454953
44.H	6.984612	4.030125	0.057105
45.B	-0.165985	3.326509	0.430945
46.O	-0.340954	3.502610	1.926927

p-Me (-6369.43)

1.C	0.083473	-1.115464	-0.702422
2.C	1.260304	-0.396146	-0.505181
3.C	1.229001	0.982438	-0.218768
4.C	-0.000361	1.703725	-0.148702
5.C	-1.187498	0.925632	-0.296051

6.C	-1.135973	-0.452187	-0.582517
7.H	2.221247	-0.903948	-0.555415
8.H	2.162517	2.220698	2.046428
9.H	-0.093335	2.961484	2.001976
10.H	-2.316663	2.111982	1.905049
11.H	-2.067012	-1.004140	-0.694063
12.H	0.115491	-2.180422	-0.922146
13.C	2.567020	1.612361	0.016081
14.C	3.563167	1.572625	-0.978230
15.C	4.829229	2.116613	-0.754506
16.C	5.157762	2.713163	0.474701
17.C	4.173384	2.733284	1.474554
18.C	2.902302	2.193728	1.251938
19.H	3.332852	1.123433	-1.941582
20.H	5.574121	2.079883	-1.547749
21.H	7.307478	2.751976	0.208642
22.H	4.403311	3.173027	2.443437
23.C	-2.566225	1.490388	-0.145837
24.C	-3.492385	1.415478	-1.203216
25.C	-4.793498	1.901377	-1.059285
26.C	-5.228532	2.468173	0.150447
27.C	-4.310749	2.529715	1.210389
28.C	-3.005238	2.049476	1.067774
29.H	-3.178416	0.991637	-2.154285
30.H	-5.480653	1.846331	-1.901989
31.H	-6.709414	3.749822	1.076154
32.H	-4.619059	2.959764	2.161684
33.B	-0.045446	3.378200	-0.014418
34.O	-0.105149	3.758042	1.451367
35.H	-2.024367	3.635356	-0.415974
36.O	-1.189175	4.001862	-0.745024
37.O	1.112793	4.057918	-0.668630
38.H	1.941273	3.728952	-0.287017
39.C	-6.647331	2.968025	0.311743
40.H	-7.035870	3.370448	-0.630208
41.H	-7.315734	2.151975	0.619377
42.C	6.519269	3.332765	0.700638
43.H	6.557522	4.349937	0.286850
44.H	6.752494	3.401797	1.768259

H (-5632.78)

1.C	0.086834	-1.124651	-0.630754
2.C	1.262337	-0.398503	-0.452404
3.C	1.228037	0.986220	-0.198576
4.C	-0.001822	1.707022	-0.142802
5.C	-1.186725	0.923363	-0.273974
6.C	-1.133625	-0.460625	-0.527496
7.H	2.224191	-0.905371	-0.492124
8.H	2.152531	2.264974	2.039815
9.H	-0.106092	3.007309	1.982877
10.H	-2.314543	2.136521	1.905120
11.H	-2.063687	-1.016474	-0.626550
12.H	0.120717	-2.194456	-0.824535
13.C	2.566192	1.621865	0.021869
14.C	3.556187	1.564343	-0.978121
15.C	4.825119	2.110734	-0.766885
16.C	5.135934	2.717539	0.456150
17.C	4.167931	2.767970	1.465298
18.C	2.896704	2.224347	1.250329
19.H	3.317718	1.102059	-1.932999

20.H	5.569994	2.066366	-1.558278
21.H	6.122479	3.143798	0.621380
22.H	4.401270	3.228071	2.422763
23.C	-2.567039	1.488539	-0.137431
24.C	-3.487423	1.388208	-1.198446
25.C	-4.793167	1.868642	-1.066076
26.C	-5.211032	2.449981	0.137253
27.C	-4.312855	2.541347	1.206239
28.C	-3.004646	2.064322	1.070121
29.H	-3.165995	0.945374	-2.138042
30.H	-5.483294	1.792922	-1.903368
31.H	-6.226373	2.824863	0.241057
32.H	-4.629425	2.981795	2.148875
33.B	-0.049663	3.384099	-0.041503
34.O	-0.108340	3.792345	1.415828
35.H	-2.030584	3.644463	-0.436266
36.O	-1.194694	3.990134	-0.784725
37.O	1.106780	4.051776	-0.710087
38.H	1.935994	3.750596	-0.308308

p-F (-5645.32)

1.C	0.086862	-1.122735	-0.640840
2.C	1.262257	-0.397251	-0.459566
3.C	1.227451	0.986844	-0.203617
4.C	-0.001605	1.708471	-0.149685
5.C	-1.186015	0.924380	-0.279006
6.C	-1.133469	-0.459128	-0.534336
7.H	2.223974	-0.904443	-0.497801
8.H	2.144414	2.266571	2.040603
9.H	-0.103784	3.018714	1.972447
10.H	-2.305757	2.144571	1.904321
11.H	-2.063527	-1.015233	-0.631628
12.H	0.120669	-2.192034	-0.836939
13.C	2.564299	1.622793	0.022252
14.C	3.558473	1.564653	-0.973085
15.C	4.829771	2.106660	-0.764531
16.C	5.096083	2.699332	0.463455
17.C	4.156619	2.769089	1.483452
18.C	2.888879	2.223979	1.252519
19.H	3.328312	1.104682	-1.930207
20.H	5.593356	2.074814	-1.536256
21.F	6.351637	3.239902	0.679885
22.H	4.408823	3.232235	2.432809
23.C	-2.565393	1.489980	-0.137167
24.C	-3.491011	1.385701	-1.192833
25.C	-4.798951	1.861087	-1.063527
26.C	-5.171545	2.433435	0.146260
27.C	-4.302674	2.545853	1.223324
28.C	-2.996637	2.067710	1.071338
29.H	-3.178278	0.942771	-2.134389
30.H	-5.510058	1.794245	-1.881732
31.F	-6.464144	2.908315	0.284556
32.H	-4.637305	2.990697	2.155754
33.B	-0.048675	3.386063	-0.054743
34.O	-0.106645	3.800394	1.400799
35.H	-2.029870	3.649136	-0.451601
36.O	-1.192740	3.989766	-0.800998
37.O	1.107120	4.050518	-0.727433
38.H	1.937223	3.754949	-0.324232

p-OCF_3 (-6707.43)

1.C	-0.002753	-1.033624	-0.782465
2.C	1.191883	-0.359805	-0.539286
3.C	1.196912	1.007770	-0.204974
4.C	-0.007697	1.766925	-0.128743
5.C	-1.213045	1.032720	-0.331342
6.C	-1.202123	-0.334851	-0.665070
7.H	2.136924	-0.896144	-0.590617
8.H	2.121218	2.125619	2.121448
9.H	-0.129429	2.939424	2.070703
10.H	-2.361657	2.151011	1.892592
11.H	-2.147673	-0.851142	-0.816499
12.H	-0.000115	-2.090633	-1.038422
13.C	2.549872	1.583610	0.074944
14.C	3.556134	1.542060	-0.908582
15.C	4.839445	2.029383	-0.652727
16.C	5.111447	2.556565	0.605793
17.C	4.155554	2.593390	1.615028
18.C	2.874778	2.104243	1.341361
19.H	3.326088	1.137832	-1.890316
20.H	5.612386	2.003542	-1.414469
21.F	8.016448	4.525391	0.991886
22.H	4.407298	2.988597	2.594108
23.C	-2.575238	1.638604	-0.194742
24.C	-3.463009	1.643010	-1.286966
25.C	-4.751702	2.167929	-1.170163
26.C	-5.153358	2.675753	0.061232
27.C	-4.313604	2.680739	1.169410
28.C	-3.024861	2.155895	1.034096
29.H	-3.132262	1.251424	-2.244777
30.H	-5.427700	2.191580	-2.019152
31.F	-8.602308	3.236284	0.569526
32.H	-4.657171	3.085987	2.115928
33.B	-0.001641	3.436714	0.074577
34.O	-0.080758	3.755541	1.551532
35.H	-1.963529	3.817066	-0.330817
36.O	-1.105493	4.125118	-0.658241
37.O	1.192073	4.102457	-0.525844
38.H	1.998694	3.788872	-0.090634
39.O	-6.447886	3.284911	0.170572
40.C	-7.478273	2.480312	0.525014
41.F	-7.694482	1.451775	-0.357922
42.F	-7.312116	1.896103	1.756304
43.O	6.444259	3.000951	0.897923
44.C	6.724029	4.303312	0.649880
45.F	6.569265	4.650217	-0.669366
46.F	5.940629	5.173407	1.366262

p-CF_3 (-6425.09)

1.C	0.079766	-1.135244	-0.639309
2.C	1.257960	-0.412829	-0.465596
3.C	1.226293	0.969910	-0.202325
4.C	0.000809	1.694717	-0.131157
5.C	-1.184248	0.913932	-0.265280
6.C	-1.138090	-0.468439	-0.528079
7.H	2.218267	-0.921319	-0.515799
8.H	2.164406	2.240072	2.036032
9.H	-0.095784	2.980884	2.006526
10.H	-2.306669	2.130468	1.917882
11.H	-2.069858	-1.020949	-0.627878

12.H	0.109776	-2.203806	-0.838914
13.C	2.564121	1.602716	0.011024
14.C	3.546737	1.547742	-0.993535
15.C	4.813056	2.098844	-0.796627
16.C	5.121727	2.706992	0.425102
17.C	4.163570	2.753152	1.447944
18.C	2.899987	2.203938	1.239557
19.H	3.307723	1.085638	-1.947273
20.H	5.550592	2.059859	-1.591301
21.F	7.340783	3.158960	-0.371645
22.H	4.401971	3.213102	2.402718
23.C	-2.560007	1.483064	-0.125091
24.C	-3.480957	1.385432	-1.186459
25.C	-4.778017	1.874533	-1.059431
26.C	-5.185237	2.465170	0.146122
27.C	-4.293615	2.551219	1.220023
28.C	-2.993227	2.061829	1.080811
29.H	-3.165665	0.939728	-2.125615
30.H	-5.467693	1.804949	-1.895833
31.F	-6.833165	3.648237	1.428982
32.H	-4.606676	2.995331	2.158927
33.B	-0.042298	3.373194	-0.015934
34.O	-0.095750	3.768020	1.442298
35.H	-2.025390	3.696765	-0.363276
36.O	-1.187155	3.984310	-0.754274
37.O	1.113408	4.040752	-0.684560
38.H	1.939791	3.797364	-0.241806
39.C	-6.591072	2.978906	0.261527
40.F	-6.918524	3.844206	-0.763106
41.F	-7.527084	1.961132	0.195238
42.C	6.467005	3.328214	0.666297
43.F	6.380696	4.691872	0.884390
44.F	7.087432	2.817034	1.789656

m-F (-5645.62)

1.C	0.085851	-1.122817	-0.623947
2.C	1.261393	-0.396750	-0.447906
3.C	1.226013	0.987779	-0.194887
4.C	-0.002061	1.710084	-0.135694
5.C	-1.185527	0.925972	-0.270203
6.C	-1.134007	-0.458132	-0.522628
7.H	2.223176	-0.903218	-0.488641
8.H	2.169715	2.277458	2.054941
9.H	-0.098547	2.992002	2.000905
10.H	-2.330045	2.169490	1.911514
11.H	-2.064131	-1.013330	-0.622185
12.H	0.119294	-2.192779	-0.815763
13.C	2.565943	1.621350	0.018477
14.C	3.553307	1.561864	-0.983774
15.C	4.823391	2.105764	-0.775955
16.C	5.148907	2.714353	0.442467
17.C	4.166063	2.748601	1.422661
18.C	2.892532	2.222648	1.248458
19.H	3.311912	1.100999	-1.937455
20.H	5.567942	2.062527	-1.566537
21.H	6.128430	3.144624	0.627548
22.F	4.468483	3.332232	2.640772
23.C	-2.567021	1.489224	-0.141633
24.C	-3.487085	1.376078	-1.201495
25.C	-4.794417	1.851974	-1.073615

26.C	-5.225118	2.444186	0.119951
27.C	-4.306689	2.532471	1.157569
28.C	-2.998921	2.074587	1.063384
29.H	-3.164326	0.927218	-2.136604
30.H	-5.486278	1.768008	-1.907581
31.H	-6.235653	2.821506	0.243298
32.F	-4.713035	3.101169	2.352255
33.B	-0.048654	3.387549	-0.022674
34.O	-0.104319	3.780285	1.438322
35.H	-2.031314	3.683394	-0.389933
36.O	-1.193372	3.999932	-0.759146
37.O	1.106266	4.058711	-0.688637
38.H	1.935598	3.781559	-0.271336

Table S4. Dihedral angles φ (in degrees, as shown in Figure 1), distance between H of OH and center of the ring (d, in Å), distance between B and center of the ring (b, in Å), and asymmetric O-H···π stretching frequency shifts (OH st, in cm^{-1}) of the 2,6-diaryl-phenylboronic acid and its boronate form in water.

				boronic acid					boronate	
**compd**	**$φ_1$**	**$φ_2$**	**d**	**b_1**	**b_2**	**O-H st**	**φ**	**d**	**b**	**O-H st**
***p*-OMe**	49.7	50.7	3.600	3.934	3.902	3684.6	62.3	2.531	4.100	3691.1
***p*-Me**	50.7	51.6	3.578	3.922	3.889	3684.6	61.9	2.542	4.106	3694.7
**H**	51.1	52.6	3.562	3.917	3.884	3684.9	62.1	2.550	4.102	3699.7
***p*-F**	51.1	52.3	3.571	3.910	3.879	3685.0	62.0	2.552	4.096	3706.8
***p*-OCF_3**	51.1	52.4	3.554	3.910	3.874	3685.7	62.0	2.563	4.095	3713.3
***p*-CF_3**	51.0	51.4	3.589	3.908	3.888	3685.9	61.7	2.564	4.088	3715.6
***m*-F**	51.6	51.5	3.606	3.912	3.882	3685.5	62.1	2.563	4.102	3709.4

Figure S5. Relative energy ΔE (in kcal mol^{-1}) of 2,6-diaryl-phenylboronic acid **H** (only φ_1 dihedral angle is varied, whereas φ_2 is kept at 52.6º) and its boronate form (only φ_1 dihedral angle is varied, whereas φ_2 is kept at 62.1º)[a] as a function of the rotation of the dihedral angle φ_1 (in degrees) in water.

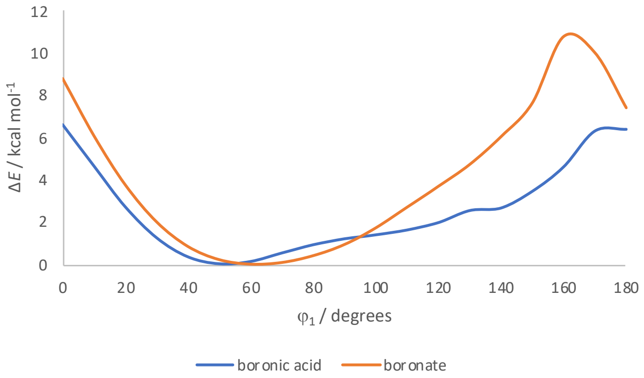

[a] The rotational barrier calculated for 2,6-diaryl-phenylboronate **H** in the range 0–180º shows how the staggered conformation is higher in energy due to the steric repulsion.

Table S5. Boronate formation energies of the 2,6-diaryl-phenylboronic acids in water and in vacuo based on the reaction $ArB(OH)_2 + 2H_2O \rightarrow ArB(OH)_3^- + H_3O^+$.

compd		water		gas	
		$\Delta E^{boronate}$	$\Delta G^{boronate}$	$\Delta E^{boronate}$	$\Delta G^{boronate}$
**1**	**H**	47.1	59.0	170.7	181.9
**2**	***p*-OMe**	47.8	61.0	174.1	185.2
**3**	***p*-Me**	47.4	59.4	172.1	183.2
**4**	***p*-F**	47.1	58.9	165.8	177.0
**5**	***p*-OCF$_3$**	47.3	59.2	160.3	171.3
**6**	***p*-CF$_3$**	47.0	58.9	158.6	169.7
**7**	***m*-F**	47.0	58.9	165.9	177.1

Figure S6. Dependence of calculated boronate formation energies Δ*E* on the Hammett sigma values (2σ) of *para*-substituted 2,6-diaryl-phenylboronic acids in a) water and b) in vacuo; based on the reaction $ArB(OH)_2 + 2H_2O \rightarrow ArB(OH)_3^- + H_3O^+$.

a)

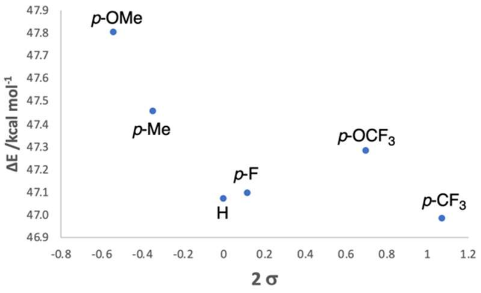

b)

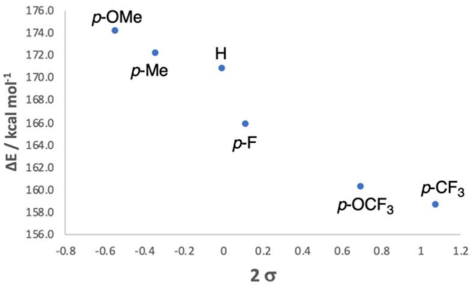

Table S6. Brønsted acidity of the 2,6-diaryl-phenylboronic acids in water and in vacuo computed as $ArB(OH)_2 \rightarrow ArB(OH)O^- + H^+$ (in kcal mol^{-1}).

		water	gas
**compd**		ΔE^{PA}	ΔE^{PA}
**1**	**H**	180.6	353.0
**2**	***p*-OMe**	181.2	353.9
**3**	***p*-Me**	181.1	354.0
**4**	***p*-F**	180.3	348.4
**5**	***p*-OCF$_3$**	180.6	343.7
**6**	***p*-CF$_3$**	180.2	342.5
**7**	***m*-F**	180.0	348.8

Table S7. Energy decomposition analysis (in kcal mol^{-1}) of the interaction between the $HB(OH)_2$ group and the π-ring in the model system (Figure 5) derived from the 2,6-diaryl-phenylboronic acids.[a]

	boronic acid					boronate				
	ΔE_{int}	ΔE_{Pauli}	ΔV_{elstat}	ΔE_{oi}[b]	ΔE_{disp}	ΔE_{int}	ΔE_{Pauli}	ΔV_{elstat}	ΔE_{oi}	ΔE_{disp}
**H**	2.30	16.27	-6.08	-3.39	-4.50	-0.54	22.93	-9.35	-8.61	-5.51
***p*-OMe**	2.04	16.58	-6.54	-3.46	-4.53	0.14	23.46	-8.88	-8.87	-5.56
***p*-Me**	2.17	16.44	-6.30	-3.43	-4.54	-0.01	23.24	-8.87	-8.82	-5.56
***p*-F**	2.49	16.25	-5.88	-3.38	-4.50	-2.60	22.91	-11.35	-8.64	-5.52
***p*-OCF_3**	2.66	16.03	-5.48	-3.35	-4.53	-5.09	22.76	-13.24	-9.03	-5.57
***p*-CF_3**	2.71	16.00	-5.42	-3.35	-4.52	-5.57	22.64	-13.38	-9.24	-5.59
***m*-F**	2.45	16.09	-5.80	-3.37	-4.47	-2.46	22.72	-10.85	-8.80	-5.53

[a] Computed at the ZORA-BLYP-D3BJ/TZ2P level in gas on equilibrium geometries in water. [b] Overlap between empty $2p_z$ of B and π of aryl decreases from 0.040 to 0.021 from *p*-OMe to *p*-CF_3.

Table S8. Energy decomposition analysis (in kcal mol^{-1}) of the interaction between the $B(OH)_2$ group and the π-ring for *p*-OMe and *p*-CF_3 based on two different approaches: a) $HB(OH)_2$···Ar (2 fragments), and b) Ar···$HB(OH)_2$···Ar (3 fragments).[a]

a) $HB(OH)_2$···Ar

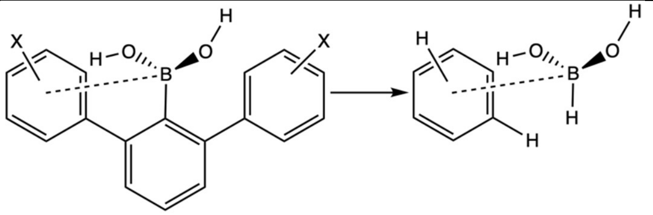

		ΔE_{int}	ΔE_{Pauli}	ΔV_{elstat}	ΔE_{oi}	ΔE_{disp}
boronic acid	***p*-OMe**	2.04	16.58	-6.54	-3.46	-4.53
	***p*-CF_3**	2.71	16.00	-5.42	-3.35	-4.52
boronate	***p*-OMe**	0.14	23.46	-8.88	-8.87	-5.56
	***p*-CF_3**	-5.57	22.64	-13.38	-9.24	-5.59

b) Ar···$HB(OH)_2$···Ar

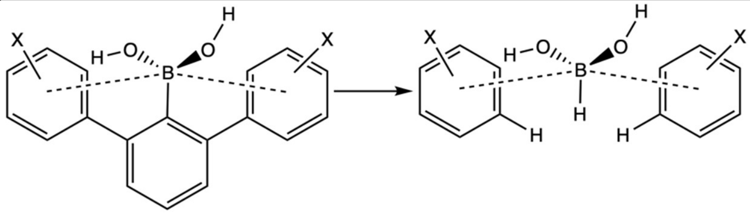

		ΔE_{int}	ΔE_{Pauli}	ΔV_{elstat}	ΔE_{oi}	ΔE_{disp}
boronic acid	***p*-OMe**	3.74	33.28	-13.02	-6.99	-9.53
	***p*-CF_3**	5.15	32.07	-10.46	-6.94	-9.52
boronate	***p*-OMe**	1.09	47.22	-17.16	-17.41	-11.57
	***p*-CF_3**	-9.87	45.59	-26.42	-17.42	-11.62

[a] Tha discussion in the manuscript has been based on the 2 fragments model system. Comparison to the 3 fragments model systems shows that the trend is the same, but only values are almost twice those for the 2 fragments model.

Table S9. Interaction energy in vacuo (ΔE_{int}^{gas}) and in aqueous solvation (ΔE_{int}^{aq}), interaction energy difference due to solvation ($\Delta\Delta E_{int}^{solv}$), desolvation of fragments (ΔE_{desolv}^{frag}) and solvation of complex (ΔE_{solv}^{comp}) energies (in kcal mol^{-1}) corresponding to the interaction between the $B(OH)_2$ group and the π-ring in the model system (Figure 5) derived from the 2,6-diaryl-phenylboronic acids and their boronate form.[a,b]

	boronic acid					boronate				
	ΔE_{int}^{aq}	ΔE_{int}^{gas}	$\Delta\Delta E_{int}^{solv}$	ΔE_{desolv}^{frag}	ΔE_{solv}^{comp}	ΔE_{int}^{aq}	ΔE_{int}^{gas}	$\Delta\Delta E_{int}^{solv}$	ΔE_{desolv}^{frag}	ΔE_{solv}^{comp}
**H**	2.70	2.30	0.40	-8.10	-7.70	3.04	-0.54	3.58	-74.17	-70.59
***p*-OMe**	2.71	2.04	0.67	-9.97	-9.30	3.28	0.14	3.14	-76.21	-73.07
***p*-Me**	2.69	2.17	0.52	-8.07	-7.55	3.13	-0.01	3.14	-74.18	-71.04
***p*-F**	2.76	2.49	0.27	-8.99	-8.72	3.12	-2.60	5.72	-75.05	-69.33
***p*-OCF_3**	2.86	2.66	0.20	-9.50	-9.30	3.36	-5.09	8.45	-75.47	-67.02
***p*-CF_3**	2.78	2.71	0.07	-9.57	-9.50	3.26	-5.57	8.83	-75.43	-66.60
***m*-F**	2.69	2.45	0.24	-9.02	-8.78	3.08	-2.46	5.54	-74.98	-69.44

[a] $\Delta E_{int}^{aq} = \Delta E_{int}^{gas} - \Delta E_{desolv}^{frag} + \Delta E_{solv}^{comp}$; $\Delta\Delta E_{int}^{solv} = \Delta E_{int}^{aq} - \Delta E_{int}^{gas}$. [b] Charge of boronate fragment in vacuo (in a.u.): -0.893, -0.890, -0.886, -0.885, -0.874 and -0.868 for *p*-OMe, *p*-Me, H, *p*-F, *p*-OCF3 and *p*-CF3, respectively.
